# Effects of open-label placebos across populations and outcomes: an updated systematic review and meta-analysis of randomized controlled trials

**DOI:** 10.1038/s41598-025-14895-z

**Published:** 2025-08-15

**Authors:** Johannes C. Fendel, Carl Tiersch, Paul Sölder, Jens Gaab, Stefan Schmidt

**Affiliations:** 1 Department of Psychosomatic Medicine and Psychotherapy, Faculty of Medicine, Medical Center - University of Freiburg, University of Freiburg, Hauptstrasse 8, 79104 Freiburg, Germany; 2 Division of Clinical Psychology and Psychotherapy, Faculty of Psychology, University of Basel, Basel, Switzerland; 3 Cognition, Action and Sustainability Unit, Department of Psychology, University of Freiburg, Freiburg, Germany

**Keywords:** Open-label placebo, Objective outcome, Meta-analysis, Systematic-review, Psychology, Medical research, Outcomes research

## Abstract

This work synthesizes and updates findings from previous systematic-reviews and meta-analyses on open-label placebos (OLPs). For the first time, it directly tests whether OLPs differ in effects between clinical and non-clinical samples, and between self-report and objective outcomes. We searched eight databases up to November 9, 2023, and included 60 randomized controlled trials (RCTs), compromising 63 separate comparisons. OLPs yielded a small positive effect across various health-related outcomes (k = 63, *n* = 4554, SMD = 0.35, CI 95% = 0.26; 0.44, *p* < 0.0001, I^2^ = 53%). The effect differed between clinical (k = 24, *n* = 1383, SMD = 0.47) and non-clinical samples (k = 39, *n* = 3171, SMD = 0.29; Q = 4.25, *p* < 0.05), as well as between self-reported (k = 55, *n* = 3919, SMD = 0.39) and objective outcomes (k = 17, *n* = 1250, SMD = 0.09; Q = 7.24, *p* < 0.01). Neithter the level of suggestiveness nor the type of control moderated the effect. These findings confirm that OLPs are effective for both clinical and non-clinical samples—particularly when effects are assessed via self-reports.

**Supplementary Information:**

The online version contains supplementary material available at 10.1038/s41598-025-14895-z.

## Introduction

The placebo effect is a well-known psychological phenomenon that can lead to significant improvements in both clinical and non-clinical populations for self-report outcomes (e.g., self-rated questionnaires) and objective outcomes (e.g., physiological, or behavioral variables). Placebos have been shown to have beneficial effects in health-related outcomes and conditions such as allergies, anxiety, Alzheimer’s disease, Parkinson’s disease, depression, fatigue, pain, as well as on physical performance and various physiological systems^1–3^. However, traditional placebos, which involve deceiving recipients about their treatment, raise ethical concerns and limit their applicability in clinical and general practice^2^. An alternative to deceptive placebos is the use of open-label placebos (OLPs), where recipients are informed transparently that they are receiving a placebo with no pharmacologically active ingredients^4^. Since the pioneering OLP trial by Kaptchuk et al^5^., numerous studies have explored various aspects of OLPs^6^.

Previous meta-analyses have demonstrated beneficial effects of OLP interventions, particularly in clinical populations and for self-report outcomes. While Spille et al.^7^ found a moderate OLP effects in non-clinical samples for self-reports, Charlesworth et al.^8^ and von Wernsdorff et al.^9^ reported moderate to large effects of OLPs in clinical samples. Buergler et al.^10^ conducted the first network meta-analyses in the field, examining how OLP effects vary depending on treatment expectation, comparator, administration route, and population. Their findings suggest that OLPs are generally more effective in clinical than non-clinical samples, that positive treatment expectations play a key role, and that the type of comparator influences effect sizes—while the route of administration had no substantial impact.

These findings highlight the need to consider both the population type (clinical vs. non-clinical) and the form of outcome (self-report vs. objective) when evaluating the effectiveness of OLPs. However, none of the existing meta-analyses statistically integrated or directly compared clinical and non-clinical samples, nor did they analyze differences between self-report and objective outcomes. To date, no meta-analysis has quantified the overall effect of OLPs across both population type and outcome forms in a single model.

A key concept in OLP research is the induction of treatment expectations through verbal suggestions in OLP interventions^11^. Most OLP interventions are accompanied by a standardized rationale originally developed by Kaptchuk et al.^12^, which typically includes the following four suggestive elements: (1) the placebo effect is powerful, (2) the body is automatically responding to placebos, (3) a positive attitude towards palcebos is not necessary, and (4) adherence to the placebo regime is important. Initial meta-analytic evidence suggests that OLPs delivered with such a suggestive rationale tend to be more effective than those without it. For example, Buergler et al.^10^ found that OLP interventions with a suggestive rationale were more effective than those without, though the available data were limited. Spille et al.^7^ reported that suggestiveness influenced objective outcomes in non-clinical populations but had no clear effect on self-reports. Taken together, these findings suggest that building expectations is a key factor in OLP effectiveness—but it remains unclear when and for which outcomes suggestive rationales actually improve treatment effects.

The type of control condition plays a crucial role in determining the observed effect size of OLP interventions. This is well established in psychotherapy research, where comparisons to waiting list (WL) control groups often yield larger effect sizes than comparisons to no treatment (NT)^13^. Buergler et al.^10^ found that OLPs in clinical samples were more effective than NT and WL, but not more effective than treatment as usual (TAU). In non-clinical samples, Spille et al. introduced the concept of covert placebo (CP)^7^—a condition where participants receive the same treatment as in the OLP group, but with a rationale designed to divert attention away from the placebo related expectations (e.g., participants are given a technical explanation for receiving the placebo treatment). Unlike deceptive placebos, CPs aime to avoid creating specific expectations in recipients about the treatment’s outcome. CPs were proposed as a potential alternative to NT, aiming to minimize expectation effects without deception. However, the limited data available from Spille et al. necessitates a reevaluation of their findings^7^.

In summary, to address these unresolved questions, we conducted a systematic review and meta-analysis that builds on and extends previous work in the field^7,9,10,14^. We derived the following research questions (RQs): First, do OLP interventions offer greater benefits compared to control groups across various outcomes and populations (RQ1)? Second, do these benefits differ between clinical and non-clinical populations (RQ2)? Third, do these benefits differ between self-report and objective outcomes (RQ3)? Fourth, does the level of expectation induction through suggestion influence the OLP effect (RQ4)? Fifth, do effect sizes vary based on the type of control group used (RQ5)?

## Methods

This systematic review and meta-analysis utilized the same databases and search terms as Spille et al.^7^ and von Wernsdorff et al.^9^. The study report adhered to the revised Preferred Reporting Items for Systematic Reviews and Meta-Analyses (PRISMA) 2020 statement^15^ (Supplemental digital appendix A1). We pre-registered the study with PROSPERO (CRD42023421961) prior to data collection.

### Eligibility criteria

We included randomized controlled trials (RCTs) that investigated the effects of OLPs on various health-related outcomes in both clinical and non-clinical populations. The OLP intervention could include any form of substance without a pharmacologically active ingredient, such as placebo pills, capsules, nasal sprays, dermatological creams, injections, acupuncture, or verbal suggestions^16^. It was essential that the OLP was administered transparently, with recipients fully informed that they were receiving a placebo^6,16^. OLPs had to be compared with one of the following control groups: CP, NT, TAU, or WL control group. We included only trials reporting outcomes on a continuous scale, whether self-report (e.g., self-rated questionnaire) or objective measure (i.e., physiological, or behavioral variables). In accordance with the Cochrane Handbook for Systematic Reviews^17^we included crossover trials only if data from the initial phase of the trial (i.e., prior to the crossover) were available. We contacted authors, if this data was not reported. If the authors could not provide the data or did not respond, we excluded the trial from the analyses.

### Information sources and search strategy

This study builds upon and updates two previous systematic reviews on open-label placebos^7,9^, which served as primary sources and starting points for identifying eligible studies. In addition, we conducted a comprehensive systematic database search on November 9, 2023, including EMBASE via Elsevier, MEDLINE via PubMed, APA PsycINFO and PSYNDEX Literature with PSYNDEX Tests via EBSCO, the Web of Science Core Collection, and the most recent edition of the Cochrane Central Register of Controlled Trials (CENTRAL, The Cochrane Library, Wiley). We used the search terms identical to those employed by von Wernsdorff et al.^9^ and Spille et al.^7^, focusing on variants of ‘open-label placebo’ (e.g., ‘placebo’, ‘open-label’, ‘non-blind’, and ‘without deception’). The number of hits for each search term in these databases is provided in Supplemental digital appendix A2. We restricted our search to publications from January 2020 onward (i.e., the search date of von Wernsdorff et al., 2021). For non-clinical samples, we included trials published from April 15, 2021 (i.e., the search data of Spille et al., 2023). Additionally, we screened all entries in the *Journal of Interdisciplinary Placebo Studies* database (JIPS, https://jips.online/) from January 2020, as well as the complete database in the Program in Placebo Studies & Therapeutic Encounter (PiPS, http://programinplacebostudies.org/), using keywords in the publication titles (e.g. ‘analgesia’, ‘expectation’, ‘non-deceptive’, ‘open-label’, ‘placebo’, ‘suggestion’). No restrictions were applied regarding the language of publication or the age of participants.

### Selection process

Results from the literature databases, including hits from the JIPS and PiPS databases, were exported into the systematic review management software Rayyan^18^. Duplicates were removed using Rayyan’s semi-automatized duplicate-detection feature. Two researchers (C.T. and P.S.) independently assessed study eligility. First, they screened all titles and abstracts, followed by a full-text assessment of reports deemed potentially eligible in the first stage by one of the investigators (see Supplemental digital appendix A3 for excluded full-texts with reasons for exclusion). Disagreements were resolved through discussion, with the supervision of two additional researchers (J.C.F. and S.S.).

### Data items and collection process

Two researchers (C.T. and P.S.) independently extracted data from the included studies in a standardized Excel form, which was piloted with three records. Data extraction covered the following areas: study details (i.e., author, year, title), sample characteristics (i.e., type of population, population size, distribution of participants), intervention and control groups (e.g., pill, cream, spray, duration), outcomes (i.e., baseline, post-intervention or change scores), and outcome form (i.e., self-report or objective). We extracted data for the outcome defined as primary outcome in the respective report. If a primary outcome was not specified, we extracted all outcomes related to the OLP intervention to minimize bias from selective outcome choice based on effect size and hypothesis fit^19^. In trials where baseline outcome scores were unavailable prior to experimental exposure, we extracted post-intervention values only.

Participants were classified into clinical populations if they met a medical condition (e.g., allergic rhinitis, cancer-related fatigue, chronic low back pain, menopausal hot flashes) or a mental disorder (e.g., major depressive disorder), as diagnosed by a clinician or psychologist^20^. Those classified as non-clinical populations were generally healthy individuals. Subclinical traits, such as test anxiety or low levels of well-being, did not qualify participants for the clinical population and were therefore categorized as non-clinical. The degree of suggestiveness of the treatment rationale was independently evaluated by two researchers (C.T. and P.S.) based on the description of the OLP administration. Rationals of OLP interventions featuring one or more elements from Kaptchuk et al. (2010, see Introduction)^12^ were rated as having a ‘high’ degree of suggestiveness, while those lacking elements of suggestive expectation induction were rated as having a ‘low’ degree of suggestiveness. In studies with different treatment rationales across separate intervention groups, both groups were extracted as distinct trials and coded accordingly as OLP+ (‘high’ degree of suggestiveness) and OLP- (‘low’ degree of suggestiveness). To ensure comparability across clinical trials, all control groups (i.e., NT, TAU, WL, and CP) were checked for compliance with given definitions of comparators^21^. Thus, NT refers to a group in which no alternative treatment is provided, while TAU included access to standard treatment practices for the condition. If participants were offered an OLP intervention following the OLP group, the control group was labeled as a WL group. For non-clinical trials, NT refers no intervention, and CP involved the same physical treatment as the OLP group, but with a rationale designed to avoid creating specific expectations regarding the outcome^7^. Missing values were addressed by contacting the authors. If the authors did not responed or could not provide the data, the study was excluded. All extracted data were cross-checked using Excel’s data validation feature. Discrepancies were resolved through discussion and consensus, supervised by (J.C.F. and S.S.).

The study at hand is an extended update of the reviews by von Wernsdorff et al.^9^ and by Spille et al.^7^. Therefore, one reviewer (C.T.) re-evaluated and extracted data from the studies included in these reviews. In cases of discrepancies regarding selection and extraction due to methodological differences (e.g., von Wernsdorff et al.^9^ extracted consistently post-intervention values only), a second reviewer (P.S.) independently re-assessed these reports. In cases where changes were necessary, both reviewers (C.T. and P.S.) independently extracted any additional data. The same data cross-checking process, as illustrated above, was applied to ensure accuracy.

### Risk of bias assessment

We assessed the risk of bias of the included trials using the Cochrane Risk of Bias tool (RoB 2.0)^22^. The RoB 2 assesses bias arising from the randomization process (domain 1), deviations from intended interventions (domain 2), missing outcome data (domain 3), measurement of the outcome (domain 4), and selection of the reported result (domain 5)^22^. The results of the five domains are aggregated into an overall risk of bias rating, which is equivalent to the worst rating in any of the domains. In line with the previous meta-analyses on OLPs^7,9,10^, we applied the same special rules to the RoB 2 to account for the unblinded nature of OLPs. When a rating of a ‘high’ risk of bias in domain 4 resulted only due to the signaling question 4.5 (‘Is it likely that the assessment of the outcome was influenced by knowledge of the intervention received?’), we overrode the suggestion of the algorithm for this domain and labeled it with ‘some concerns’. Since eliciting treatment expectation is a crucial mode of action of OLPs, the effect of knowing about the group allocation cannot be separated from the placebo or nocebo effect (i.e., excitement or disappointment respectively)^9^. Moreover, we would have lost all variance in the assessment, as all studies would have received a ‘high’ overall rating as consequence. The RoB 2 for the newly included studies was assessed by two researchers independently (C.T. and P.S.), with discrepancies resolved through discussion and consensus. The results from the risk of bias assessments of the studies included in previous versions of the review by Spille et al. (2023)^7^ and von Wernsdorff et al.(2021)^9^ and the present one are presented in the Supplemental digital appendix A4.

### Data synthesis and analysis

Statistical analyses were performed using R, version 4.5.0^23^. To evaluate the effects of the OLP interventions, we calculated standardized mean differences (SMDs) by subtracting the mean pre-post change in the intervention group from the mean pre-post change in the control group and dividing the result by the pooled pre-intervention standard deviation^24^. For studies that reported only post scores, SMDs were calculated based on these values. Standard errors of the means were converted into SDs following the guidelines outlined in the Cochrane Handbook^17^. We used *Hedges’ g* which corrects for bias due to small sample sizes^25^. We interpreted values of 0.20, 0.50, and 0.80 as small, moderate, and large effect, respectively^26^. Whenever a primary outcome was clearly defined in a study, this outcome was used for effect size calculation. In studies without defined primary outcome^27–51^we computed SMDs for all relevant outcomes—whether self-report or objective—individually, and then averaged them to obtain an overall SMD estimate for the respective trial^52^. This was done by the aggregate function in R from the *metafor package*^53^under the assumption of an intra-study correlation coefficient of *ρ* = 0.6^54^. In studies where the type of administration of the OLP intervention varied (e.g., one group received OLP nasal spray and another intervention group received OLP pills)^29,38,43,55,56^the mean pre- and post-values along with their SD were aggregated prior to data analysis, in accordance with the guidelines outlined in the Cochrane Handbook^17^. We aggregated the interventions groups of these trials as follows: for Barnes et al.^56^, we combined the ‘Semi-Open Label’ and ‘Fully-Open Label’ intervention groups; for El Brihi et al.^34^, we combined the groups receiving one and four OLP pills per day; for Kube et al.^38^, we aggregated the ‘OLP-H’ (hope) and ‘OLP-E’ (expectation) groups; for Olliges et al.^43^, we combined the ‘OLP pain’ and ‘OLP mood’ groups; and for Winkler et al.^29^, we merged the ‘OLP nasal active’, ‘OLP nasal passive’, and ‘OLP capsule’ groups. In studies where the OLP rationale was manipulated accross two independent samples^32,42,57^, the SMDs were calculated separately for each intervention group (OLP + and OLP− respectively) and treated as two distinct trials. To prevent unity-of-analysis error due to double counting, the control group data was divided in half for each intervention group in these cases^17^. Similarly, if a trial contributed to more than one comparison (i.e., assessing both self-report and objective outcomes)^27,31,36,38,42,49,58^ again, the sample size was divided by the number of comparisons to prevent unit-of-analysis error due to double-counting. To ensure consistent interpretation of the direction of effects where a positive SMD value indicates a beneficial effect of the OLP intervention for recipients, the means of some studies were multiplied by − 1, as outlined in the Cochrane Handbook^17^.

After aggregating all effect sizes within studies, we conducted a meta-analysis using the *meta* package in R^59^. Anticipating heterogeneity among trials, we employed a random-effects model with the inverse-variance weighting method^60^. We weighted the trials based on post-intervention sample sizes, which are more conservative than pre-intervention samples sizes. All tests were two-tailed. Heterogeneity among studies was assessed using the *Q*-statistic and quantified with the *I*^2^ index as well as *prediction intervals*. The *I*^2^ values indicate the percentage of total variance between effects that is due to true effect variation and are interpreted as follows: 0 to 40% might not be important; 30 to 60% may represent moderate heterogeneity; 50 to 90% may represent substantial heterogeneity; and 75 to 100% is considered to constitute considerable heterogeneity^17^. The prediction intervals indicate true effect variation and represent the range into which the true effect size of all populations will fall^61^. If the prediction interval lies entirely on the positive side (i.e., does no include the zero), favoring the OLP intervention, it suggests that, despite variations in effect sizes, the OLP is likely to be beneficial; however, is important to note that broad prediction intervals are relatively common and reflect inherent variability in the data^54^.

### Sensitivity analysis

We conducted four sensitivity analyses to examine the robustness of results. First, we excluded outliers utilizing the “non-overlapping confidence intervals” approach, which regards a comparison as outlier, if its 95% confidence interval of the effect size did not overlap with the 95% confidence interval of the pooled effect size^54^. Second, we excluded studies assessed with a ‘high’ risk of bias according to the RoB 2 assessment. We chose the criterion ‘high’, because almost all studies had at least a risk to ‘some concerns’ due to the lack of blinding of the OLP interventions and to self-reported outcomes. Third, we considered potential publication bias using Duval and Tweedie’s trim and fill procedure, which provides an estimate of the pooled effect size after adjusting for asymmetry in the funnel plot^62^. Fourth, we estimated the overall OLP effect using a hierarchical three-level meta-analytic model, with effect sizes nested in studies^54^.

### Reporting bias assessment and certainty assessment

We assessed publication bias by creating a funnel plot, which plots effect estimates (SMDs) from individual studies against their standard error (SE). We visually inspected the funnel plot for asymmetry, conducted Egger’s regression test, which regresses the SMDs against their SE^63^ and calculated Rosenthal’s Fail-Safe-N^64^.

## Results

### Trial selection

**Fig. 1:**
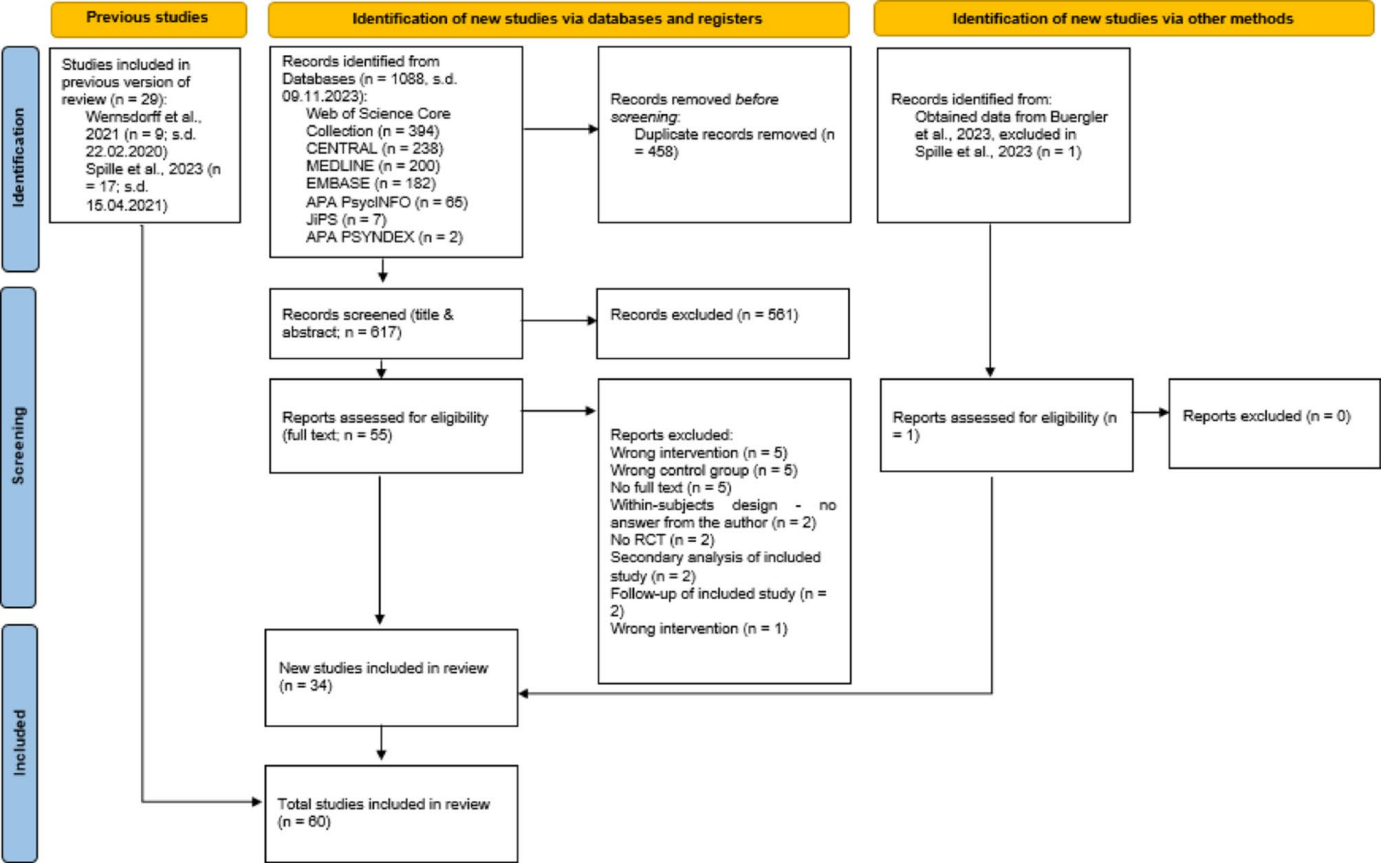
PRISMA flow diagram for study selection. In four cases, the individual article reported data from two independent experiments each, which were considered as two distinct studies.

We included 60 RCTs with 63 seperate comparisons. Of these, 9 trials were from von Wernsdorff et al.^9^, 17 from Spille et al.^7^, and 34 were identified in our updated search (for a flow chart see Fig. 1). The chance-corrected agreement between raters after the full-text screening in the updated search was substantial (κ = 0.78)^65^. We further applied a set of additional selection and exclusion steps based on eligibility criteria, data availability, trial design (e.g., follow-ups, crossover studies), and control group definitions; a detailed list of these decisions is provided in Supplemental digital appendix A5.

### Trial characteristics

Detailed qualitative characteristics of included trials are presented in Supplemental digital appendix A6, and detailed numerical characteristics are presented in Supplemental digital appendix A7. The trials were published in English between 2001 and 2023. The 60 RCTs (37 non-clinical and 23 clinical trials) involved a total of 4648 participants, with 2492 individuals randomized to an OLP intervention and 2156 individuals randomized to control groups. The mean age of participants weighted for the pre-intervention sample size was 30.5 years (SD = 8.7) with an age range from 14 to 70 years. The weighted mean percentage of females was 72%. Sample sizes ranged from 9 to 133 individuals. The duration of the trials was from one to 90 days (Mean/SD = 12.4/16.6, Median = 7). Studies were conducted in Germany^29,31,38–41,43–47,49,57,66,67,78–84^, in the USA^12,35,37,69,76,85–93^, in Australia^32,34,51,56,94^ and Austria^28,48,58,95–97^Switzerland^42,50,55,98^ in Israel^99^, Japan^27^, the Netherlands^89^, New Zealand^30^, Portugal^33^, and the United Kingdom^36^.

Non-clinical trials investigated OLP effects on experimentally induced emotional distress of various types, including distressing pictures^35,58,67,95^, experimentally induced sadness^78–80^guilt^98^, intrusive memories^40,^and distress related to social exclusion^50^; experimentally induced pain^38,42,83,90^; physical and mental well-being^32,34,44^; arousal and well-being^31,49^; psychological distress^29^; physiological processes such as nausea^56^, wound healing^30^, experimentally induced itch^89^, sleep quality^28^, caffeine withdrawal symptoms^94^, and physiological recovery^51^; cognitive performance^36,82^; test anxiety^45,55^; promoting beneficial behaviors such as Progressive Muscle Relaxation^96^ and Acts of Kindness^97^; and experimentally induced acute stress^46^.

Clinical trials investigated OLP effects on allergic rhinitis^39,41,47,57,84^, chronic low-back pain^27,33,81,85^, and major depressive disorder^48,87,99^. Additionally, trials focused on cancer-related fatigue^37,91,92^, irritable bowel syndrome^12,69,88^, the impact of OLPs on knee osteoarthritis pain^43^, and the reduction of experimentally induced emotional distress in women diagnosed with major depressive disorder^66^. Trials also included a conditioning OLP paradigm, of which two investigated the reduction of opioid medication after surgery^76,93^, and one on opioid use disorder^86^.

The type of OLP administration varied across the primary trials: 33 trials used OLP pills, 11 trials used active or passive nasal sprays (i.e., with or without a prickling sensation), four trials administered the OLP in the form of drops, three as a dermal cream, three as a decaffeinated coffee, and one each in the form of imaginary pills, oral spray, oral vapor, saline injection, sham acupuncture, sham deep brain stimulation, syrup, and verbal suggestion. Seven trials used CP as control group, 38 trials NT, six trials TAU, and nine trials WL. Three trials applied a conditioning design.

Regarding the OLP rationale, 52 trials used a treatment rationale with ‘high’ levels of suggestiveness, five trials used a treatment rationale without suggestive elements and three trials manipulated the treatment rationale, resulting in one intervention group with suggestion (OLP+) and one without (OLP−). In total, 84 self-report and 61 objective outcomes were extracted from non-clinical trials, and 29 self-report and five objective outcomes from clinical trials. The chance-corrected agreement between raters regarding the outcomes to be extracted was substantial (κ = 0.61)^65^.

### Risk of bias in studies

Supplemental digital appendix A4 presents the risk of bias assessment. In the updated review, nine trials^27–29,85,86,91,93,94,96^ (26%) were rated as having a high risk of bias. Eight of these trials had missing outcome data^27,28,85,86,91,93,94,96^, while one trial^29^ had concerns about the randomization process and one trial failed to analyze the data according to intention-to-treat^96^. Twenty-four trials (71%) were rated as having a moderate risk of bias or ‘some concerns’. This was primarily due to the fact, that the majority of the trials collected self-report data, which could be influenced by participants’ knowledge of their group allocation. In the overall sample, 12 primary trials (20%) were rated as having a ‘high’ risk of bias, 46 trials (77%) had ‘some concerns’ and two trials (3%) had a ‘low’ risk of bias.

### Effects of OLPs

**Fig. 2:**
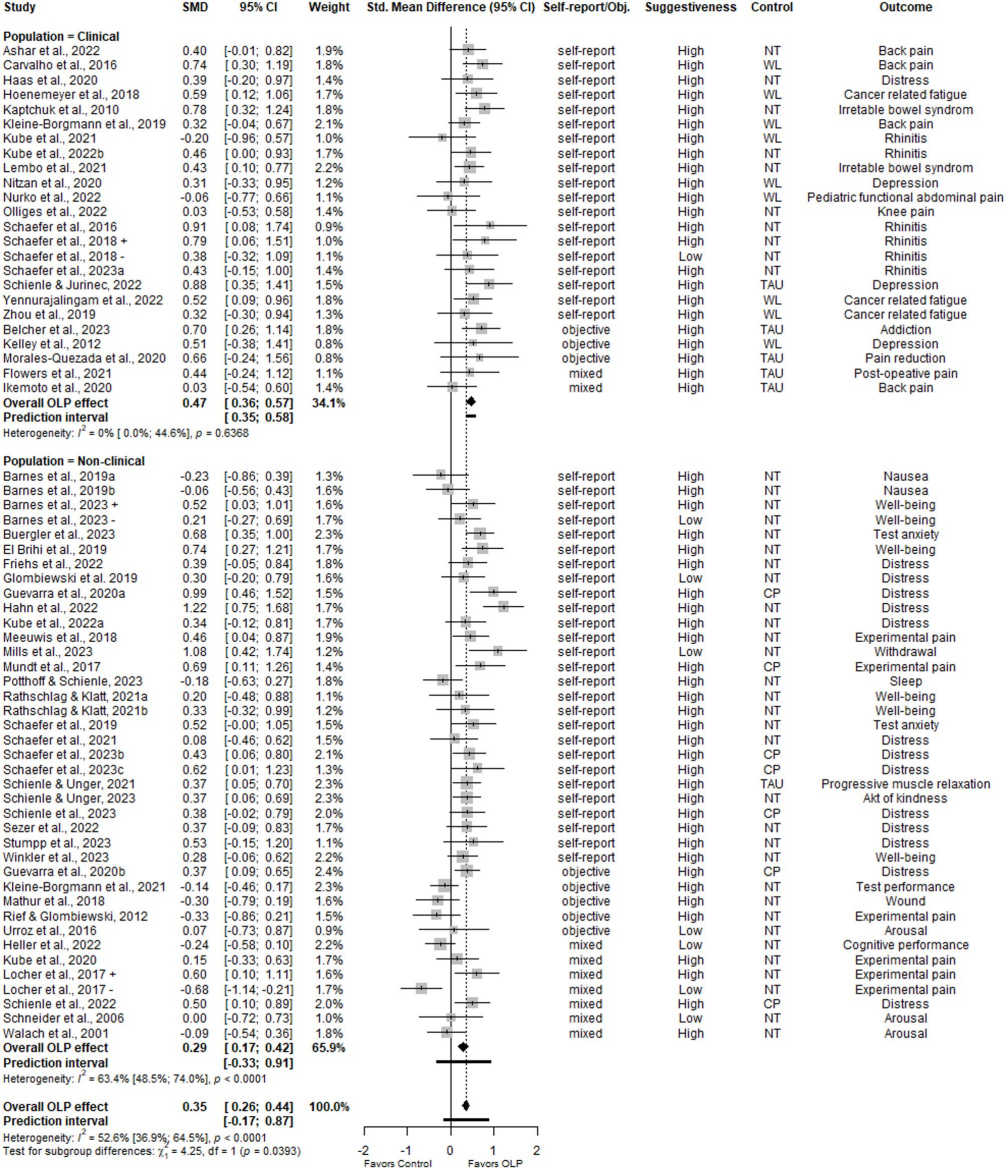
Forest plot of the effects of open-label placebos vs. control conditions on all outcomes. Studies were weighted using the inverse-variance method. The size of the grey squares indicates the weight of each study, while the whiskers represent the 95% confidence intervals. The overall SMD is shown as a black diamond. In trials employing and comparing several treatment rationals, intervention groups receiving a suggestive treatment rationale are denoted by author names and year followed by a +’, whereas those receiving a rationale without suggestion are denoted by author names and year followed by a ‘−’.

Table 1 summarizes the results of the main, sensitivity, and subgroup analyses. Figure 2 displays the forest plot for the main analysis of OLPs. We analyzed 63 comparisons across 60 trials, including three trials that manipulated treatment rationales in two distinct intervention groups (OLP + and OLP−)^32,42,57^. The meta-analysis revealed a small but significant positive effect of OLP interventions compared to control groups (k = 63, *n* = 4554, SMD = 0.35, CI 95% = 0.26; 0.44, *p* < 0.0001; I^2^ = 53%), with moderate heterogeneity between trials (RQ1). The prediction interval ranged from − 0.17 to 0.87, indicating that while the average effect favors OLPs, true effects in future settings could vary and may include no effect. However, the sensitivity analyses produced similar results, demonstrating the robustness of the results.

**Table 1:** Effects of open-label placebos (OLPs). Main, sensitivity analyses, and subgroup analyses.

Analyses	k	*N* ^a^	g	CI_lb	CI_ub	I^2^	I^2^_lb	I^2^_ub	τ	PI_lb	PI_ub	*p*	Q	p_sg
Main and sensitivity analyses														
All comparisons	63	4554	0.35	0.26	0.44	52.64	36.85	64.49	0.25	− 0.17	0.87	****		
Outliers excluded	56	3938	0.40	0.33	0.46	4.45	0.00	29.81	0.05	0.27	0.52	****		
High risk excluded	51	3651	0.32	0.22	0.42	53.82	36.55	66.39	0.26	− 0.21	0.86	****		
Adjusted for publication bias	71	5012	0.28	0.18	0.37	61.26	49.88	70.05	0.31	− 0.34	0.90	****		
3-Level Model	179	4554^b^	0.33	0.23	0.43	56.38	–	–	0.32	− 0.40	1.06	****		
Subgroup analyses														
Population														
Clinical	24	1383	0.47	0.36	0.57	0.00	0.00	44.61	0.00	0.35	0.58	****	4.25	*
Non-clinical	39	3171	0.29	0.17	0.42	63.41	48.46	74.02	0.30	− 0.33	0.91	****	39	
Form of outcome^c^														
Self-report	55	3919	0.39	0.30	0.49	50.78	32.83	63.93	0.25	− 0.11	0.90	****	7.24	**
Objective	17	1250	0.09	− 0.12	0.29	66.33	43.91	79.79	0.33	− 0.65	0.83			
Suggestiveness														
High	55	4131	0.38	0.30	0.47	42.20	20.15	58.16	0.20	− 0.03	0.80	****	2.20	
Low	8	423	0.11	− 0.25	0.46	70.31	38.40	85.69	0.42	− 0.97	1.19			
Type control														
NT	41	2967	0.29	0.17	0.41	61.09	45.36	72.29	0.30	− 0.34	0.91	****	5.43	
WL	9	517	0.41	0.24	0.59	0.00	0.00	64.80	0.00	0.21	0.62	****		
CP	7	666	0.50	0.34	0.65	0.00	0.00	70.81	0.00	0.30	0.69	****		
TAU	6	404	0.50	0.27	0.73	17.85	0.00	62.65	0.12	0.06	0.94	****		

^a^Sample sizes reflect post-intervention or change-score Ns used for variance estimation for meta-analytic weighting, and may therefore differ from baseline sample sizes reported in text.

^b^For the 3-Level Model, *n* refers to the total number of unique participants included across all studies, although multiple effect sizes per study were modeled.

^c^Study count *k* and number of participants *n* in the form of outcome subgroups do not sum to the overall total of all comparisons, as some studies included both self-report and objective outcomes. Adjusted sample sizes were used in subgroup comparisons to correct for unit-of-analysis issues in cases where such overlap occurred.

*k* number of studies, *CI* confidence interval, *lb* lower bound of 95% CI, *ub* upper bound of 95% CI, *PI* prediction interval, *sg* subgroup, NT no treatment, TAU treatment as usual, CP covert placebo, WL waiting list.

*p < 0.05; **p < 0.01; ***p < 0.001; ****p < 0.0001.

OLP interventions had a small significant effect in both clinical samples (k = 24, *n* = 1383, SMD = 0.47, CI 95% = 0.36; 0.57, *p* < 0.0001; I^2^ = 0%) and non-clinical samples (k = 39, *n* = 3171, SMD = 0.29, CI 95% = 0.17; 0.42, *p* < 0.0001; I^2^ = 63%), with lower heterogeneity in clinical samples. The effectiveness differed significantly between clinical and non-clinical samples (Q = 4.25, *p* < 0.05, RQ2). The prediction interval for clinical samples did not include zero (0.35 to 0.58), suggesting that future studies are consistently likely to show a positive effect. In contrast, the prediction interval for non-clinical samples ranged from − 0.33 to 0.91, indicating that effects may vary more widely and could include no effect in some settings.

OLP interventions significantly improved self-report outcomes (k = 55, *n* = 3919, SMD = 0.39, CI 95% = 0.30; 0.49, *p* < 0.0001; I^2^ = 51%) but showed no significant effect on objective outcomes (k = 17, *n* = 1250, SMD = 0.09, CI 95% = − 0.12; 0.29, *p* = 0.41; I^2^ = 66%), with moderate to substantial heterogeneity. The difference in effectiveness between self-report and objective outcomes was significant (Q = 7.24, *p* < 0.01, RQ3). The prediction interval for self-report outcomes ranged from − 0.11 to 0.90, suggesting the possibility of no effect in future studies. For objective outcomes, the interval ranged from − 0.65 to 0.83, clearly encompassing zero, indicating high uncertainty and that OLPs may not consistently affect objective measures.

Trials with a high suggestive treatment rationale showed a small significant OLP effect (k = 55, *n* = 4131, SMD = 0.38, CI 95% = 0.30; 0.47, *p* < 0.0001; I^2^ = 42%), whereas trials with low suggestiveness did not (k = 8, *n* = 423, SMD = 0.11, CI 95% = − 0.25; 0.46, *p* = 0.55; I^2^ = 70%). However, the difference between these subgroups based on suggestiveness was not significant (Q = 2.20, *p* = 0.14, RQ4). The prediction interval for high suggestiveness (– 0.03 to 0.80) slightly included zero, indicating small remaining uncertainty about whether effects will consistently be positive. For low suggestiveness, the interval was wider (– 0.97 to 1.19) suggesting high variability and low confidence in consistent benefit.

Finally, small to medium significant effects of OLPs were observed across all types of control groups (Table 1). The difference between control subgroups was not significant (Q = 5.43, *p* = 0.14, RQ5). Prediction intervals varied between control types: they included zero for no-treatment, but did not for treatment-as-usual (0.06 to 0.94), waitlist (0.21 to 0.62), and covert placebo (0.30 to 0.69), suggesting more robust and consistent effects of OLPs in those contexts.

### Reporting bias

**Fig. 3:**
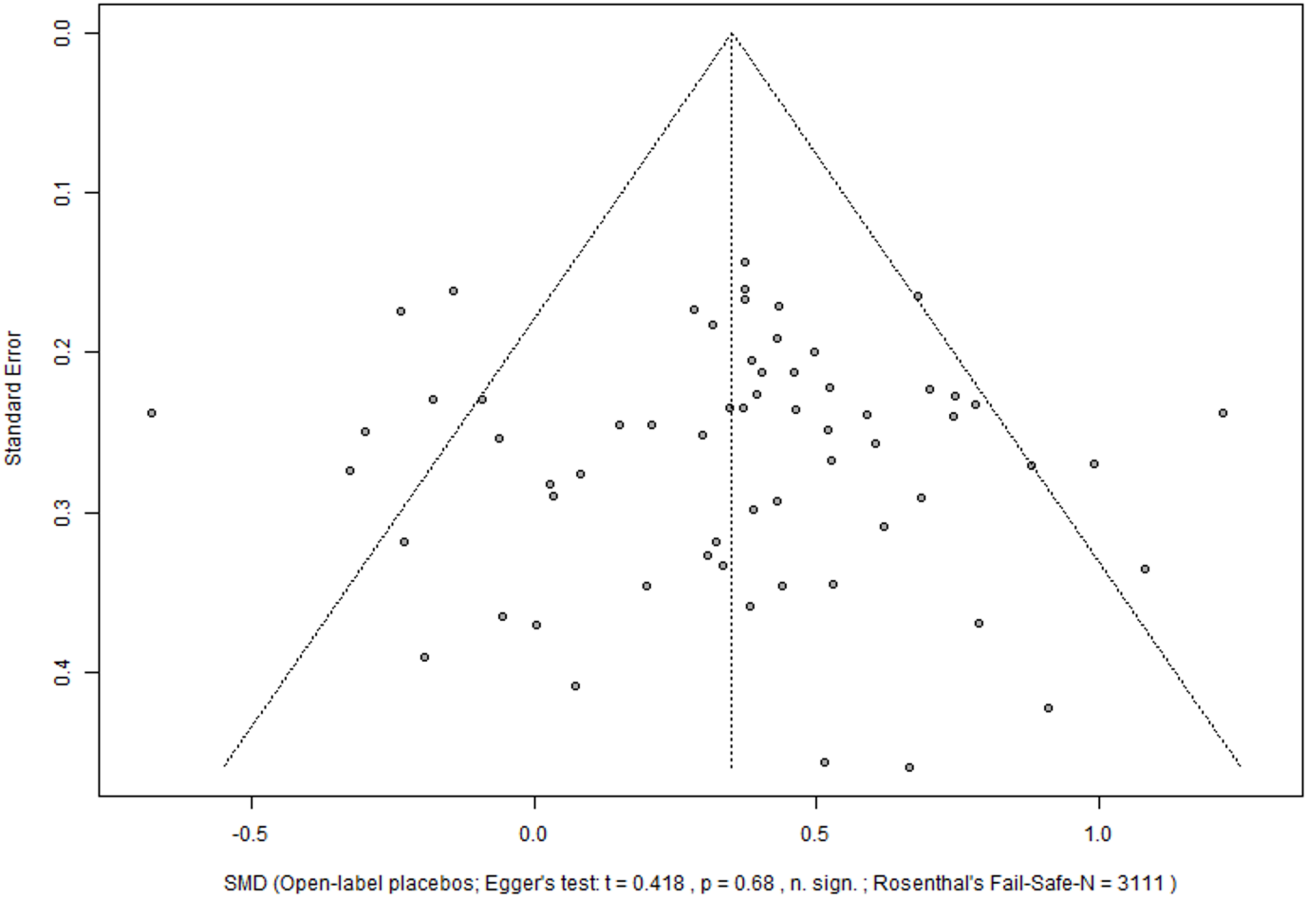
Funnel plot of meta-analysis across populations and outcomes. The funnel plot plots the effect estimates (SMDs) from individual studies against their standard error. *SMD* Standardized mean difference.

The visual inspection of the funnel plot (Fig. 3) and the Egger’s regression showed no evidence of publication bias for the main meta-analysis across outcomes and samples (t = 0.42, *p* = 0.68), the Fail-Safe-N was 3111 studies).

## Discussion

This systematic review and meta-analysis updates and synthesizes findings from four previous meta-analyses of randomized controlled trials (RCTs) on open-label placebos (OLPs)^7–10^. With the inclusion of 34 additional trials, our review includes a total of 60 trials with 4648 participants and is substantially larger than previous meta-analyses. This expansion allowed for novel statistical comparisons, offering deeper insights into OLP effects. For the first time, the review facilitated direct comparisons between self-report and objective outcomes and synthesized data from clinical and non-clinical samples. The review encompasses various OLP interventions, evaluates different outcomes, and compares these interventions across diverse control groups and sample types.

The results indicate that OLP interventions produce a small but clinically relevant positive effect across a wide range of health related outcomes compared to control groups (SMD = 0.35; RQ1). In addition, OLPs had a small effect in both clinical and non-clinical samples, with a significant difference in effectiveness between these populations (RQ2). OLPs improved self-report outcomes but had no significant effect on objective outcomes, with a significant difference between the two (RQ3). OLPs were effective when the treatment rationale was highly suggestive (i.e., inducing treatment expectations) but not when it was not, though the difference between these groups was not significant (RQ4). Lastly, OLPs exhibited small to medium significant effects against all kinds of control groups, with no significant differences between these control subgroups (RQ5). The overall heterogeneity was moderate in the primary analysis, but decreased substantially when excluding outliers, focusing solely on clinical samples, or comparing OLPs to TAU, covert placebos or waiting list controls. Consistently, prediction intervals in these subsets did not include zero, suggesting more reliable and generalizable effects in those conditions despite remaining variability elsewhere.

To our knowledge, this systematic review and meta-analysis is the most comprehensive synopsis of OLP research to date. The overall positive effect of OLPs (RQ1) aligns with previous meta-analyses^7–10^, confirming with extensive data that placebos are effective even when recipients are aware they are receiving a treatment, which is not pharmacologically active^4^. This finding suggests that OLPs can serve as a viable alternative to deceiving participants at least in certain clinical and research settings^2^.

Although OLP interventions benefit both clinical and non-clinical samples, they affect these groups differently (RQ2). Our meta-analysis shows a larger effect in clinical samples (SMD = 0.47) compared to non-clinical samples (SMD = 0.29). This difference likely arises from the greater need for relief in clinical samples due to disease-related impairments, which provides a larger potential for improvement^101,102^. For example, Barnes et al.^32^ found in a non-clinical sample larger OLP effects on wellbeing only for participants whose baseline scores were below the average, suggesting that higher distress levels might enhance OLP effects. Additionally, clinical trials lasted longer than to non-clinical trials, with a mean duration of 23.5 days (SD = 21.6) compared to 5.5 days (SD = 6.2) for non-clinical samples. This extended duration may allow patients to develop a stronger connection to the healing ritual aspects of OLPs compared to non-clinical samples^6^. It may also leave more time for the intervention to unfold. The results for clinical samples align with findings of Buergler et al.^10^, who reported an almost identical effect for OLP pills compared to NT (SMD = 0.46). This effect is somewhat smaller than those suggested by earlier meta-analyses on OLPs in clinical samples (SMD = 0.88^8^ and SMD = 0.72^9^). However, the earlier meta-analyses were based on a relatively small body of evidence with small sample sizes. The reduction in magnitude of the effect sizes may indicate a time-lag bias, where significant results were more likely to be reported early in OLP research, while later studies with insignificant results were likely to be published over time^10^. Similar earlier studies may have lower methodological quality. A sensitivity analysis excluding trials with a high risk of bias in Wernsdorff revealed a reduced effects size of SMD = 0.49 being very close to our finding^9^. The beneficial effects of OLP interventions in non-clinical samples (SMD = 0.29) are smaller than in clinical samples. They are also smaller than in the prior meta-analysis by Spille et al.^7^ as well as the significant findings for studies applying nasal spray in non-clinical samples from Buergler et al.^10^. This may be due to the combination of studies with self-reported and objective outcome which were separated in Spille et al. (2023). With respect to Buergler et al. (2023) also methodological differences may account for these variations. For example, they excluded trials with balanced-placebo designs as well as those with CP as a control group^10^, while our analysis included such trials (see eligibility criteria). In addition, they included non-randomized trials^74,75^, which we excluded. Moreover, Buergler et al. (2023) used a different classification for clinical and non-clinical samples^10^, based on the investigated states/conditions rather than on the health status of the participants/patients. Therefore they classified some trials as clinical, which were considered to be non-clinical by our approach^34,45,82,96^. Lastly, Buergler et al. included studies with deceptive placebo control group^10^, which were excluded in our analysis. These smethodological differences resulted in fewer trials in the non-clinical network compared to our analysis (12 trials in Buergler et al.^10^ vs. 37 in the present analysis).

OLPs appear to affect self-report and objective outcomes differently (RQ3). While OLPs have a beneficial effect on self-report outcomes across both clinical and non-clinical populations, they show no effect on objective outcomes. Despite increasing the number of trials (from 17 to 55 comparisons for self-report outcomes and from eight to 17 comparisons for objective outcomes), the effect sizes remain broadly consistent with those reported by Spille et al. (2023) (self-report SMDs: 0.43 vs. 0.39; objective SMDs: − 0.02 vs. 0.09). The results suggest that OLPs have a positive impact on individuals’ perceptions of their health, but that there is currently no meta-analytical proof that they do also alter biological or behavioral variables. Nevertheless, this finding corresponds to meta-analytical findings on deceptive placebos in clinical trials, in which patient-reported outcomes also demonstrate larger effect sizes (SMD = 0.26) than observer-rated outcomes (SMD = 0.13), although here the latter are significant^103^. The non-significant SMD of 0.09 in the present meta-analysis may indicate either that OLPs have no effect on objective outcomes or that there are currently too few studies to reveal a significant effect.

In the history of placebo research, deceptive placebos were initially thought to be merely suggestive to the patient, with no corresponding biological effect. This picture changed when the endogenous opoid system was identified as one biological mechanism of placebo analgesia^104,105^. Today, there is a large body of literature that clearly demonstrates the biological mechanisms and effects of deceptive placebos^106^. Whether the research on OLPs will take the same avenue remains to be seen. To date there exists to our knowledge only one experimental study demonstrating a biological mechanisms of OLP analgesia^107^ as well as some more fMRI studies on healthy volunteers reporting neurobiological changes^67,95,108^. Our finding that the effect size for objective measures of OLPs is not significant, unlike the one for deceptive placebos, highlights the need for further investigations into the psychological and biological mechanisms behind OLP effects. As more trials become available in the future, we will be able to break down the category of objective outcomes by type of variable (e.g. biological, behavioral, etc.) and time-span (e.g. short-term vs. long-term). Of particular interest are also the variables suggestion and expectation, especially with regard to interpersonal differences. Another fruitful focus could be the perspective to understand OLPs as a healing ritual and in particular addressing its embodied aspects^109–111^.

The degree of suggestiveness in the treatment rationale accompanying the administration of OLPs in our review had an impact on their effectiveness (RQ4). Trials with a suggestive rationale that aimed to build treatment expectations showed a small positive effect (SMD = 0.38), while those without such a rationale did not demonstrate significant effects (SMD = 0.11). These results align with Buergler at al. (2023), emphasizing that building expectancy is crucial for OLP effectiveness and that merely administering an inert treatment is not sufficient^10^. This supports the prevailing view that OLP effects are based on the elicitation of treatment expectations through a suggestive rationale^6,112^.

Regarding the impact of different types of control groups on OLP effectiveness (RQ5), we compared OLP interventions against four types of comparators: 41 used no treatment controls (NT), nine used weight list (WL) controls, seven used a covert placebo (CP), and six used treatment as usual (TAU). The inferential analysis revealed no significant differences (*p* = 0.14) among these control groups, indicating that OLPs yield beneficial effects across all control types. However, as “absence of evidence is not evidence of absence”^113^, these findings should be interpreted carefully. Moreover, these findings somewhat contrast with Buergler et al., who concluded that OLPs performed better than ‘nothing’ but only slightly better than ‘something’ and that comparator groups should be chosen carefully^10^. As for population types, methodological differences between our study and Buergler et al.^10^ may account for these discrepancies in results (see above).

The presented systematic review and meta-analysis has several strengths. First, it adhered to rigorous methodology, including a pre-registered search protocol and data synthesis, with two independent researchers assessing eligibility, extracting data, and evaluating risk of bias. Second, it incorporated 23 additional RCTs compared to the most recent previous meta-analysis of OLPs^10^, thereby strengthening the robustness of the results. Third, we directly compared differences in the effectiveness of OLPs between self-report and objective outcomes as well as between clinical and non-clinical samples and these differences yielded valuable insights. Finally, we conducted multiple sensitivity analyses, which confirmed the robustness of the main findings and reinforced observed trends in OLP effects.

Several limitations should be considered when interpreting the results. First, most of the trials included in the review had relatively small sample sizes (i.e., fewer than 100 participants). As already denoted by Buergler et al., this limitation raised the possibility of a so-called ‘small study effect’^10^, where smaller trials often report larger treatment effects than larger trials^63,114,115^. Second, we combined effect sizes across different populations (e.g., across various clinical conditions) and forms of outcomes (e.g., self-report vs. objective), which could bias the observed OLP effect, as various populations and outcomes may respond differently to OLP administration. In a similar vein, we independently aggregated multiple treatment arms with varying degree of suggestiveness in the treatment rationale (i.e. OLP + and OLP−) and analyzed these arms separately in the analysis. This approach may potentially skew effect sizes due to over-representation and inter-correlation of outcomes and treatment arm in question. However, we tried to avoid over-representation in terms of unit-of-analysis error due to double counting by splitting the sample sizes of the respective control groups. Moreover, we tried to model the potential dependency of multiple effect size estimates for one sample by conducting a sensitivity analysis using a hierarchical three-level meta-analytic model, with effect sizes nested in studies, leading to comparable results for the main analysis. Third, we did not peform a GRADE rating. This is because, we consider the GRADE system to be suboptimal for the use in our OLP review. We see especially the inability to blind patients/participants to the intervention, affecting the risk of bias rating, and the inclusion of a huge varieties of different outcomes in our review as problematic with respect to GRADE.

Our systematic review has identified several research gaps. First, trials with larger sample sizes are essential to enhance the robustness of the findings. Second, longer study durations are required, as most trials in non-clinical populations lasted only one day, and as long-term follow-up trials on OLP interventions in clinical samples have yielded mixed results^72,73^. This raises questions about the durability of OLP effects and underscores the need for further research on long-term effects in both clinical and non-clinical settings. Second, to increase the validity of the results, future trials should include more representative samples. Non-clinical trials in particular, often included younger and predominantly female participants compared to clinical populations. Third, as OLPs affect self-report and objective outcomes differently, future research should put a stronger focus on objective outcomes, particularly in clinical settings. Finally, this systematic review and meta-analysis included several trials published by the same research groups, indicating the need for research teams with less allegiance to OLPs to mitigate potential bias.

In summary, the results of the presented systematic review and meta-analysis indicate several key findings: (1) OLP interventions demonstrate small but significant positive overall benefit across various populations and outcomes in a larger sample of studies. (2) OLPs are more effective in clinical than in non-clinical samples. (3) OLPs are effective on self-report measures but their effect is unclear for objective measures. This finding raises important research questions about their biological and psychological mechanisms, particularly in comparison to deceptive placebos. (4) The suggestiveness of the treatment rationale in OLP interventions is crucial, as trials lacking suggestive elements did not yield significant beneficial effects. (5) Different comparator groups produced similar estimates of OLP effectiveness.

## Supplementary Information

Below is the link to the electronic supplementary material.

Supplementary Material 1

## Data Availability

All data generated or analysed during this study are either included in this published article and its supplementary information file or have been deposited at https://osf.io/pxzcg/.
