# Supplementary material for: Effects of open-label placebos across populations and outcomes: an updated systematic review and meta-analysis of randomized controlled trials
Source: Sci Rep. 2025 Aug 15;15:29940. doi: 10.1038/s41598-025-14895-z (PMC12356945; doi:10.1038/s41598-025-14895-z)
Supplement: Supplementary file 1 — Supplementary Material 1 [file 41598_2025_14895_MOESM1_ESM.docx]

**Supplemental digital appendix**

**Table of contents**

[Supplemental digital appendix A1: PRISMA 2020 checklist. 2](#_Toc203988318)

[Supplemental digital appendix A2: Search Strategy for EMBASE via Elsevier, for MEDLINE via PubMed, for PsycINFO via EBSCO, for PSYNDEX Literature with PSYNDEX Tests via EBSCO, for Web of Science Core Collection, for the Cochrane Central Register of Controlled Trials (CENTRAL) 5](#_Toc203988319)

[Supplemental Table A3: Excluded records in full-text screening with reasons for exclusion. 11](#_Toc203988320)

[Supplemental digital appendix A4: Within studies’ risk of bias assessment for included RCTs on five RoB 2.0 criteria and overall rating. 13](#_Toc203988321)

[Supplemental digital appendix A5: Supplementary information on study inclusion, exclusion, and data handling. 16](#_Toc203988322)

[Supplemental digital appendix A6: Qualitative characteristics of included trials. 17](#_Toc203988323)

[Supplemental digital appendix A7: Numerical characteristics of included trials 31](#_Toc203988324)

[References 41](#_Toc203988325)

## Supplemental digital appendix A1: PRISMA 2020 checklist.

| **Section and Topic** | **Item #** | **Checklist item** | **Location where item is reported** |
| --- | --- | --- | --- |
| **TITLE** | | | **Page** |
| Title | 1 | Identify the report as a systematic review. | Title page, Abstract |
| **ABSTRACT** | | |  |
| Abstract | 2 | See the PRISMA 2020 for Abstracts checklist. | Abstract |
| **INTRODUCTION** | | |  |
| Rationale | 3 | Describe the rationale for the review in the context of existing knowledge. | 3-5 |
| Objectives | 4 | Provide an explicit statement of the objective(s) or question(s) the review addresses. | 5 |
| **METHODS** | | |  |
| Eligibility criteria | 5 | Specify the inclusion and exclusion criteria for the review and how studies were grouped for the syntheses. | 5 |
| Information sources | 6 | Specify all databases, registers, websites, organizations, reference lists and other sources searched or consulted to identify studies. Specify the date when each source was last searched or consulted. | 5-6 |
| Search strategy | 7 | Present the full search strategies for all databases, registers and websites, including any filters and limits used. | Supplemental digital appendix |
| Selection process | 8 | Specify the methods used to decide whether a study met the inclusion criteria of the review, including how many reviewers screened each record and each report retrieved, whether they worked independently, and if applicable, details of automation tools used in the process. | 6 |
| Data collection process | 9 | Specify the methods used to collect data from reports, including how many reviewers collected data from each report, whether they worked independently, any processes for obtaining or confirming data from study investigators, and if applicable, details of automation tools used in the process. | 6-8 |
| Data items | 10a | List and define all outcomes for which data were sought. Specify whether all results that were compatible with each outcome domain in each study were sought (e.g. for all measures, time points, analyses), and if not, the methods used to decide which results to collect. | 6-8 |
|  | 10b | List and define all other variables for which data were sought (e.g. participant and intervention characteristics, funding sources). Describe any assumptions made about any missing or unclear information. | 6-8 |
| Study risk of bias assessment | 11 | Specify the methods used to assess risk of bias in the included studies, including details of the tool(s) used, how many reviewers assessed each study and whether they worked independently, and if applicable, details of automation tools used in the process. | 8 |
| Effect measures | 12 | Specify for each outcome the effect measure(s) (e.g. risk ratio, mean difference) used in the synthesis or presentation of results. | 9-10 |

| Synthesis methods | 13a | Describe the processes used to decide which studies were eligible for each synthesis (e.g. tabulating the study intervention characteristics and comparing against the planned groups for each synthesis (item #5)). | 9-10 |
| --- | --- | --- | --- |
|  | 13b | Describe any methods required to prepare the data for presentation or synthesis, such as handling of missing summary statistics, or data conversions. | 8-10 |
|  | 13c | Describe any methods used to tabulate or visually display results of individual studies and syntheses. | 14, Figure 2, Table 1 |
|  | 13d | Describe any methods used to synthesize results and provide a rationale for the choice(s). If meta-analysis was performed, describe the model(s), method(s) to identify the presence and extent of statistical heterogeneity, and software package(s) used. | 9-10 |
|  | 13e | Describe any methods used to explore possible causes of heterogeneity among study results (e.g. subgroup analysis, meta-regression). | 4-5, (Research questions) |
|  | 13f | Describe any sensitivity analyses conducted to assess robustness of the synthesized results. | 10-11 |
| Reporting bias assessment | 14 | Describe any methods used to assess risk of bias due to missing results in a synthesis (arising from reporting biases). | 10-11 |
| Certainty assessment | 15 | Describe any methods used to assess certainty (or confidence) in the body of evidence for an outcome. | n/a |
| **RESULTS** | | |  |
| Study selection | 16a | Describe the results of the search and selection process, from the number of records identified in the search to the number of studies included in the review, ideally using a flow diagram. | 11, Figure 1 |
|  | 16b | Cite studies that might appear to meet the inclusion criteria, but which were excluded, and explain why they were excluded. | Supplemental digital appendix |
| Study characteristics | 17 | Cite each included study and present its characteristics. | 12, Supplemental digital appendix |
| Risk of bias in studies | 18 | Present assessments of risk of bias for each included study. | Supplemental digital appendix |
| Results of individual studies | 19 | For all outcomes, present, for each study: (a) summary statistics for each group (where appropriate) and (b) an effect estimate and its precision (e.g. confidence/credible interval), ideally using structured tables or plots. | Figure 2 |
| Results of syntheses | 20a | For each synthesis, briefly summaries the characteristics and risk of bias among contributing studies. | Table 1, Supplemental digital appendix |
|  | 20b | Present results of all statistical syntheses conducted. If meta-analysis was done, present for each the summary estimate and its precision (e.g. confidence/credible interval) and measures of statistical heterogeneity. If comparing groups, describe the direction of the effect. | Table 1 |
|  | 20c | Present results of all investigations of possible causes of heterogeneity among study results. | Table 1, Supplemental digital appendix |
|  | 20d | Present results of all sensitivity analyses conducted to assess the robustness of the synthesized results. | Table 1 |
| Reporting biases | 21 | Present assessments of risk of bias due to missing results (arising from reporting biases) for each synthesis assessed. | Supplemental digital appendix |
| Certainty of evidence | 22 | Present assessments of certainty (or confidence) in the body of evidence for each outcome assessed. | n/a |
| **DISCUSSION** | | |  |
| Discussion | 23a | Provide a general interpretation of the results in the context of other evidence. | 15-19 |
|  | 23b | Discuss any limitations of the evidence included in the review. | 18-19 |
|  | 23c | Discuss any limitations of the review processes used. | 18-19 |
|  | 23d | Discuss implications of the results for practice, policy, and future research. | 19 |
| **OTHER INFORMATION** | | |  |
| Registration and protocol | 24a | Provide registration information for the review, including register name and registration number, or state that the review was not registered. | 5 |
|  | 24b | Indicate where the review protocol can be accessed, or state that a protocol was not prepared. | 5 |
|  | 24c | Describe and explain any amendments to information provided at registration or in the protocol. | n.a. |
| Support | 25 | Describe sources of financial or non-financial support for the review, and the role of the funders or sponsors in the review. | n.a. |
| Competing interests | 26 | Declare any competing interests of review authors. | 29 |
| Availability of data, code and other materials | 27 | Report which of the following are publicly available and where they can be found: template data collection forms; data extracted from included studies; data used for all analyses; analytic code; any other materials used in the review. | 29 |

*Notes.* This checklist has been adapted from Table 1 in Page, M. J., McKenzie, J. E., Bossuyt, P. M., Boutron, I., Hoffmann, T. C., Mulrow, C. D., Shamseer, L., Tetzlaff, J. M., Akl, E. A., Brennan, S. E., Chou, R., Glanville, J., Grimshaw, J. M., Hróbjartsson, A., Lalu, M. M., Li, T., Loder, E. W., Mayo-Wilson, E., McDonald, S., … Moher, D. (2021). The PRISMA 2020 statement: An updated guideline for reporting systematic reviews. Systematic Reviews*, 10(*1*),* 89*.* <https://doi.org/10.1186/s13643-021-01626-4>*.*

## Supplemental digital appendix A2: Search Strategy for EMBASE via Elsevier, for MEDLINE via PubMed, for PsycINFO via EBSCO, for PSYNDEX Literature with PSYNDEX Tests via EBSCO, for Web of Science Core Collection, for the Cochrane Central Register of Controlled Trials (CENTRAL)

***Search strategy in EMBASE via Elsevier***

| Step | Query | Results |
| --- | --- | --- |
| #1 | placebo:ti,ab,kw AND [2020-2024]/py | 65,581 |
| #2 | placebos:ti,ab,kw AND [2020-2024]/py | 583 |
| #3 | placebo*:ti AND [2020-2024]/py | 12,797 |
| #4 | #2 OR #3 | 13,129 |
| #5 | (told OR nondecept* OR 'non decept*' OR nonconceal* OR 'non conceal*' OR nonblind* OR 'non blind*' OR 'without deception' OR 'without conceal*' OR 'without blind*':ti,ab) AND [2020-2024]/py | 3,933 |
| #6 | #4 AND #5 | 58 |
| #7 | ((open OR 'open label') NEAR/1 placebo*) AND [2020-2024]/py | 152 |
| #8 | #6 OR #7 | 182 |

***Search strategy in MEDLINE via PubMed***

| Step | Query | Publication date | Results |
| --- | --- | --- | --- |
| #1 | placebos[Title/Abstract] | from 2020/1/1 - 3000/12/12 | 484 |
| #2 | placebo*[Title] | from 2020/1/1 - 3000/12/12 | 9,187 |
| #3 | #1 OR #2 | from 2020/1/1 - 3000/12/12 | 9,456 |
| #4 | told[Title/Abstract] OR nondecept*[Title/Abstract] OR "non decept*"[Title/Abstract] OR nonconceal*[Title/Abstract] OR "non conceal*" [Title/Abstract] OR nonblind*[Title/Abstract] OR "non blind*"[Title/Abstract] OR unblind*[Title/Abstract] OR "without deception"[Title/Abstract] OR "without conceal*"[Title/Abstract] OR "without blind*"[Title/Abstract] | from 2020/1/1 - 3000/12/12 | 3,739 |
| #5 | #3 AND #4 | from 2020/1/1 - 3000/12/12 | 99 |
| #6 | "open placebo*"[Title/Abstract] OR "open label placebo*"[Title/Abstract] | from 2020/1/1 - 3000/12/12 | 118 |
| #7 | #5 OR #6 | from 2020/1/1 - 3000/12/12 | 200 |

***Search strategy in APA PsycINFO via EBSCO***

| Step | Query | Limiters | Results |
| --- | --- | --- | --- |
| #1 | TI Placebos OR AB Placebos | Published date:  20200101 | 4,413 |
| #2 | TI Placebo* | Published date:  20200101 | 930 |
| #3 | # 1 OR #2 | Published date:  20200101 | 284,105 |
| #4 | TI (told OR nondecept* OR "non decept*" OR nonconceal* OR "non conceal*" OR nonblind* OR "non blind*" OR unblind* OR "without deception" OR "without conceal*" OR "without blind*") OR AB (told OR nondecept* OR "non decept*" OR nonconceal* OR "non conceal*" OR nonblind* OR "non blind*" OR unblind* OR "without deception" OR "without conceal*" OR "without blind*") | Published date:  20200101 | 1,640 |
| #5 | #3 AND #4 | Published date:  20200101 | 23 |
| #6 | TI ((open OR “open label”) N1 Placebo*) OR AB ((open OR “open label”) N1 Placebo*) | Published date:  20200101 | 44 |
| #7 | #5 OR #6 | Published date:  20200101 | 65 |

***Search strategy in APA PSYNDEX Literature with PSYNDEX Tests via EBSCO***

| Step | Query | Limiters | Results |
| --- | --- | --- | --- |
| #1 | TI Placebos OR AB Placebos | Published date:  20200101 | 136 |
| #2 | TI Placebo* | Published date:  20200101 | 132 |
| #3 | # 1 OR #2 | Published date:  20200101 | 12,585 |
| #4 | TI (told OR nondecept* OR "non decept*" OR nonconceal* OR "non conceal*" OR nonblind* OR "non blind*" OR unblind* OR "without deception" OR "without conceal*" OR "without blind*") OR AB (told OR nondecept* OR "non decept*" OR nonconceal* OR "non conceal*" OR nonblind* OR "non blind*" OR unblind* OR "without deception" OR "without conceal*" OR "without blind*") | Published date:  20200101 | 62 |
| #5 | #3 AND #4 | Published date:  20200101 | 2 |
| #6 | TI ((open OR “open label”) N1 Placebo*) OR AB ((open OR “open label”) N1 Placebo*) | Published date:  20200101 | 31 |
| #7 | #5 OR #6 | Published date:  20200101 | 2 |

***Search strategy in Web of Science Core Collection***

| Step | Query | Timespan | Results |
| --- | --- | --- | --- |
| #1 | placebos[Title/Abstract] | 2020-01-01 to 3000-12-12 | 484 |
| #2 | placebo*[Title] | 2020-01-01 to 3000-12-12 | 9,187 |
| #3 | #1 OR #2 | 2020-01-01 to 3000-12-12 | 9,456 |
| #4 | told[Title/Abstract] OR nondecept*[Title/Abstract] OR "non decept*"[Title/Abstract] OR nonconceal*[Title/Abstract] OR "non conceal*" [Title/Abstract] OR nonblind*[Title/Abstract] OR "non blind*"[Title/Abstract] OR unblind*[Title/Abstract] OR "without deception"[Title/Abstract] OR "without conceal*"[Title/Abstract] OR "without blind*"[Title/Abstract] | 2020-01-01 to 3000-12-12 | 3,739 |
| #5 | #3 AND #4 | 2020-01-01 to 3000-12-12 | 99 |
| #6 | "open placebo*"[Title/Abstract] OR "open label placebo*"[Title/Abstract] | 2020-01-01 to 3000-12-12 | 118 |
| #7 | #5 OR #6 | 2020-01-01 to 3000-12-12 | 200 |

***Search Strategy in The Cochrane Central Register of Controlled Trials (CENTRAL)***

| Step | Query | Limiters | Results |
| --- | --- | --- | --- |
| #1 | (placebos):ti,ab,kw | Publication Year from 2020 to present | 1203 |
| #2 | (placebo*):ti | Publication Year from 2020 to present | 15244 |
| #3 | # 1 OR #2 | Publication Year from 2020 to present | 15959 |
| #4 | (told OR nondecept* OR (non NEXT decept*) OR nonconceal* OR (non NEXT conceal*) OR nonblind* OR (non NEXT blind*) OR unblind OR "without deception" OR (without NEXT conceal*) OR (without NEXT blind*)):ti,ab,kw | Publication Year from 2020 to present | 1731 |
| #5 | #3 AND #4 | Publication Year from 2020 to present | 55 |
| #6 | (open OR open NEXT label) NEAR/1 placebo*:ti,ab,kw | Publication Year from 2020 to present | 205 |
| #7 | #5 OR #6 | Publication Year from 2020 to present | 238 |

## Supplemental Table A3: Excluded records in full-text screening with reasons for exclusion.

| **Record** | **Exclusion reasons** |
| --- | --- |
| 1. Ashar, Y., Perlis, R., Liston, C., Gunning, F., & Wager, T. (2022). Effects of pain reprocessing therapy on attributed causes of chronic back pain^1^ | 6 |
| 1. Bandak, E., Christensen, R., Overgaard, A., Kristensen, L. E., Ellegaard, K., Guldberg-Møller, J., Bartholdy, C., Hunter, D. J., Altman, R., Bliddal, H., & Henriksen, M. (2022). Exercise and education versus saline injections for knee osteoarthritis: A randomised controlled equivalence trial^2^ | 2 |
| 1. Braescher, A.-K., Ferti, I.-E., & Witthöft, M. (2022). Open-label placebo effects on psychological and physical well-being: A conceptual replication study^3^ | 4 |
| 1. Bush, N. J., Boissoneault, J., Letzen, J., Staud, R., & Robinson, M. E. (2023). Task-dependent functional connectivity of pain is associated with the magnitude of placebo analgesia in pain-free individuals^4^ | 1 |
| 1. Bush, N., Robinson, M., Bryan, M., Staud, R., & Boissoneault, J. (2021). Task-dependent functional connectivity of pain-related brain regions is related to magnitude of placebo analgesia^5^ | 1 |
| 1. Carvalho, C., Pais, M., Cunha, L., Rebouta, P., Kaptchuk, T. J., & Kirsch, I. (2021). Open-label placebo for chronic low back pain: A 5-year follow-up^6^ | 7 |
| 1. Choi, D.-H., Lee, I.-S., & Chae, Y. (2020). Open label placebo: Pill and needle^7^ | 3 |
| 1. Davies, J. N., Sharpe, L., Day, M. A., & Colagiuri, B. (2022). How do placebo effects contribute to mindfulness-based analgesia? Probing acute pain effects and interactions using a randomized balanced placebo design^8^ |  |
| 1. De Vita, M. J., Maisto, S. A., Gilmour, C. E., McGuire, L., Tarvin, E., & Moskal, D. (2022). The effects of cannabidiol and analgesic expectancies on experimental pain reactivity in healthy adults: A balanced placebo design trial^9^ | 2 |
| 1. Disley, N., Kola-Palmer, S., & Retzler, C. (2021). A comparison of open-label and deceptive placebo analgesia in a healthy sample^10^ | 5 |
| 1. Frommelt, T., Traykova, M., Platt, B., & Wittekind, C. E. (2023). The influence of outcome expectancy on interpretation bias training in social anxiety: An experimental pilot study^11^ | 1 |
| 1. Guevarra, D., Kross, E., & Moser, J. S. (2023). Outsourcing affect regulation to non-deceptive placebos^12^ | 3 |
| 1. Haas, J. W., Winkler, A., Rheker, J., Doering, B. K., & Rief, W. (2022). No open-label placebo effect in insomnia? Lessons learned from an experimental trial^13^ | 5 |
| 1. Hamberger, J., Jarczok, M., Hinterberger, T., Loew, T., Meissner, K., Gündel, H., & Weimer, K. (2023). Health economic evaluation of an open-label placebo intervention in patients with functional post-covid syndrome^14^ | 3 |
| 1. Henriksen, M., Christensen, R., Kristensen, L. E., Bliddal, H., Bartholdy, C., Boesen, M., Ellegaard, K., Guldberg-Møller, J., Hunter, D. J., Altman, R., & Bandak, E. (2023). Exercise and education vs intra-articular saline for knee osteoarthritis: A 1-year follow-up of a randomized trial^15^ | 2 |
| 1. Hoenemeyer, T. W., Baidwan, N. K., Hall, K., Kaptchuk, T. J., Fontaine, K. R., & Mehta, T. S. (2021). An exploratory analysis of the association between catechol-o-methyltransferase and response to a randomized open-label placebo treatment for cancer-related fatigue^16^ | 6 |
| 1. Kleine-Borgmann, J., Dietz, T.-N., Schmidt, K., & Bingel, U. (2023). No long-term effects after a 3-week open-label placebo treatment for chronic low back pain: A 3-year follow-up of a randomized controlled trial^17^ | 7 |
| 1. Lee, S., Choi, D.-H., Hong, M., Lee, I.-S., & Chae, Y. (2022). Open-label placebo treatment for experimental pain: A randomized-controlled trial with placebo acupuncture and placebo pills^18^ | 4 |
| 1. Meeuwis, S. H., Van Middendorp, H., Lavrijsen, A. P. M., Veldhuijzen, D. S., & Evers, A. W. M. (2021). Open- And closed-label placebo and nocebo suggestions about a sham transdermal patch^19^ | 2 |
| 1. Meijer, S., Karacaoglu, M., van Middendorp, H., Veldhuijzen, D., Jensen, K., Peerdeman, K., & Evers, A. (2023). Efficacy of open-label counterconditioning for reducing nocebo effects on pressure pain^20^ | 2 |
| 1. Nurko, S., Saps, M., Kossowsky, J., Zion, S., Vaz, K., Hawthorne, K., Wu, R., Di Lorenzo, C., Ciciora, S., Rosen, J., & al, et. (2020). 814 placebo without deception is effective in the treatment of children with functional gastrointestinal disorders (fgids)^21^ | 3 |
| 1. Pan, Y., Meister, R., Löwe, B., Kaptchuk, T. J., Buhling, K. J., & Nestoriuc, Y. (2020). Open-label placebos for menopausal hot flushes: a randomized controlled trial. Scientific reports, 10(1), 20090^22^. | 8 |
| 1. Ort, S., Waibl, P., Stang, M., Funke, S. A., Dalkner, N., & Meissner, K. (2023). The effects of pilates and open-label placebo on severe primary dysmenorrhea-a randomized controlled pilot study^23^ | 3 |
| 1. van Lennep, J. (Hans) P. A., van Middendorp, H., Veldhuijzen, D. S., Peerdeman, K. J., Blythe, J. S., Thomaidou, M. A., Heyman, T., & Evers, A. W. M. (2023). The optimal learning cocktail for placebo analgesia: A randomized controlled trial comparing individual and combined techniques^24^ | 1 |
| 1. Zaworski, K., Kadłubowska, M., & Baj-Korpak, J. (2023). Impact of verbal suggestions on finger flexor activation and strength in healthy individuals^25^ | 1 |

Exclusion specification: (1) Wrong intervention (e.g., deceptive placebo), (2) Wrong control group (e.g., deceptive placebo), (3) No full text (e.g., study protocols, background (theoretical) articles), (4) Within-subject design – no answer from the author, (5) no RCT (e.g., no randomization), (6) Secondary analysis of included study, (7) Follow-up of included study, (8) missing outcome, no answer from authors.

## Supplemental digital appendix A4: Within studies’ risk of bias assessment for included RCTs on five RoB 2.0 criteria and overall rating.

*Note*. The colors represent the risk of bias, with green indicating low risk, yellow indicating some concerns, and red indicating high risk of bias. D1 = risk of bias arising from the randomization process, D2 = risk of bias due to deviations from the intended interventions, D3 = risk of bias due to missing outcome data, D4 = risk of bias in measurement of the outcome, D5 = risk of bias in selection of the reported result, Overall = overall risk of bias.

## Supplemental digital appendix A5: Supplementary information on study inclusion, exclusion, and data handling.

We included two individual studies reported in one article meeting the eligibility criteria^26^, which were excluded in Spille et al. (2023)^27^ due to missing values. Here, we obtained the necessary data from Buergler et al. (2023)^28^. One trial identified through PiPS^29^ had not been included in any prior systematic reviews. Four records reported the results of two separate experiments with independent samples, both of which were included separately in the analysis^26,30–32^. We excluded several eligible studies from the meta-analysis for specific reasons: one study previously included by Wernsdorff et al. (2021)^33^ did not meet our inclusion criteria, as the primary outcome was unavailable and the authors did not respond^22^; data from two crossover studies were unavailable^3,18^; and two studies were follow-ups of included trials^6,17^, where we used the parent trial instead. The follow-up studies were excluded because they only re-investigated the intervention group^6^, or included participants who switched to an OLP intervention after the initial trial (both studies). Other exclusions involved studies lacking randomization^13,34^ or the absence of a suitable control group^35^. Moreover, we excluded a treatment arm in Rathschlag & Klatt (2021)^31^ because the author could provide only raw outcome data. Furthermore, we excluded the previously included study by Sandler (2010)^36^ in von Wernsdorff et al. (2021)^33^ because the control group did not meet the TAU definition implemented in the present analysis.

## Supplemental digital appendix A6: Qualitative characteristics of included trials.

| Title | Authors,  Year | Country | Condition | Sample | Treatment duration in days | Type of OLP^a^ | N^IG^ (% female)^b^ | Mean age ^IG^ ^c^ (SD) ^d^ | Suggestiveness | Type of CG^e^ | N^CG^ (%female) | Mean age ^CG^ (SD) | Objective outcomes | Self-report outcomes | Rationale for outcome choice | Risk of bias |
| --- | --- | --- | --- | --- | --- | --- | --- | --- | --- | --- | --- | --- | --- | --- | --- | --- |
| Effect of pain reprocessing therapy vs placebo and usual care for patients with chronic back pain: A randomized clinical trial | Ashar et al., 2022 | USA | Chronic low back pain | Clinical | 28 | OLP saline injection | 44 (49) | 39.4 (14.9) | high | NT^f^ | 47 (54) | 41.3 (3.4) |  | Brief Pain Inventory Short Form, NRS^g^ | PO^h^ | High |
| Deceptive but not open label placebos attenuate motion-induced nausea | Barnes et al., 2019a | Australia | Exp.^i^ nausea | Non-clinical | 2 | OLP vapor (fully- & semi-open) | 29 (59.8) | 20.3 (3.3) | high | NT | 15 (46.7) | 22.9 (5.5) |  | Self-rated nausea, VAS^j^ | PO | Some concerns |
| Deceptive but not open label placebos attenuate motion-induced nausea | Barnes et al., 2019b | Australia | Exp. nausea | Non-clinical | 2 | OLP sham deep brain stimulation | 31 (67.7) | 20.1 (3.1) | high | NT | 31 (67.7) | 19.8 (2.1) |  | Self-rated nausea, VAS | PO | Some concerns |
| The role of positive information provision in open-label placebo effects | Barnes et al., 2023 | Australia | Physical and Mental Well-Being | Non-clinical | 6 | OLP pill | 35 (77.1) | 19.4 (2.0) | high | NT | 32 (62.5) | 19.9 (3.8) |  | Subjective Health Complaints (SHC)  Insomnia Severity Index (ISI)  Depression Anxiety Stress Scale (DASS-21)  Warwick-Edinburgh Mental Well-being Scale (WEMWBS) | POs | Some concerns |
|  |  |  |  |  |  | OLP-^k^ pill | 35 (54.3) | 23.4 (3.4) | low |  |  |  |  |  |  |  |
| Effectiveness of conditioned open-label placebo with methadone in treatment of opioid use disorder: A randomized clinical trial | Belcher et al., 2023 | USA | Opioid use disorder | Clinical | 90 | cOLP^l^ pill | 77 (31.2) | 47.6 (10.9) | high | TAU^m^ | 54 (42.6) | 43.5 (11.2) | Methadone dose on day 90 in milligram |  | PO | High |
| Imaginary pills and open-label placebos can reduce test anxiety by means of placebo mechanisms | Buergler et al., 2023 | Switzerland | Test anxiety | Non-clinical | 21 | OLP pill (physical & imaginary) | 114 (87.6) | 22.6 (3.9) | high | NT | 59 (85) | 23 (4.7) |  | Brief German Test Anxiety Inventory (PAF) | PO | Some concerns |
| Open-label placebo treatment in chronic low back pain: A randomized controlled trial | Carvalho et al., 2016 | Portugal | Chronic low back pain | Clinical | 21 | OLP pill | 41 (70.7) | 44.4 (13.2) | high | WL^n^ | 42 (71.4) | 44.1 (13.7) |  | Pain intensity, NRS  Roland-Morris Disability Questionnaire (RMDQ) | Longest follow-up of POs | Some concerns |
| Prescribing placebos: An experimental examination of the role of dose, expectancies, and adherence in open-label placebo effects | El Brihi et al., 2019 | Australia | Physical and Mental Well-Being | Non-clinical | 7 | OLP pill (1/day & 4/day) | 61 (80) | 20.3 (4.4) | high | NT | 27 (80) | 18.9 (2) |  | Depression Anxiety Stress Scale 21 (DASS-21)  Warwick-Edinburgh Mental Wellbeing Scale (WEMWBS)  Subjective Health Complaints inventory (SHC)  Pittsburgh Sleep Quality Index (PSQI) | Outcomes related to hypothesis of the primary study | Some concerns |
| Conditioned open-label placebo for opioid reduction after spine  surgery: A randomized controlled trial | Flowers et al., 2021 | USA | Opioid reduction after spine  surgery | Clinical | 17 | cOLP pill | 19 (37) | 59.1 (13.1) | high | TAU | 22 (59) | 61.2 (13.0) | Morphine milligram equivalents (MME) | Mini Brief Pain Inventory (mini-BPI) | All suitable for analysis | High |
| Deceptive and non-deceptive placebos to reduce sadness: A five-armed experimental study | Friehs et al., 2022 | Germany | Emotional distress - sadness | Non-clinical | 1 | OLP nasal spray (personal & scientific | 63 (69.8) | 24.6 (6.6) | high | NT | 29 (82.8) | 23.1 (4.9) |  | Positive and Negative Affect Schedule-Expanded (PANAS-X), subscale sadness | PO | Some concerns |
| Placebo mechanisms in depression: An experimental investigation of the impact of expectations on sadness in female participants | Glombiewski et al. 2019 | Germany | Emotional distress - sadness | Non-clinical | 1 | OLP- nasal spray | 32 (100) | 23.1 (3.1) | low | NT | 32 (100) | 23.9 (4) |  | Positive and Negative Affect Schedule-Expanded Form (PANAS-X), subscale sadness | Relevant for hypothesis | Some concerns |
| Placebos without deception reduce self-report and neural measures of emotional distress | Guevarra et al., 2020a | USA | Emotional distress – negative pictures | Non-clinical | 1 | OLP nasal spray | 29 (34.5) | 18.8 (0.74) | high | CP^o^ | 33 (39.4) | 18.6 (0.83) |  | Emotional distress on a nine-point Likert scale | Outcomes related to hypothesis of the primary study | High |
| Placebos without deception reduce self-report and neural measures of emotional distress | Guevarra et al., 2020b | USA | Emotional distress – negative pictures | Non-clinical | 1 | OLP nasal spray | 99 (100) | 19.8 (2.4) | high | CP | 99 (100) | 19.9 (2.1) | Sustained late positive potential (sustained LPP) |  | Outcomes related to hypothesis of the primary study | Some concerns |
| Expectation-induced placebo effect on acute sadness in women with major depression: An experimental investigation | Haas et al., 2020 | Germany | Emotional distress - sadness | Clinical | 1 | OLP nasal spray | 24 (100) | 23.0 (3.55) | high | NT | 22 (100) | 26.5 (7.12) |  | Positive and Negative Affect Schedule -Expanded (PANAS-X), subscale sadness | PO | Some concerns |
| Even when you know it is a placebo, you experience less sadness: First evidence from an experimental open-label placebo investigation | Hahn et al., 2022 | Germany | Emotional distress - sadness | Non-clinical | 1 | OLP nasal spray | 42 (100) | 23.7 (3.3) | high | NT | 42 (100) | 25.8 (7) |  | Positive and Negative Affect Schedule-Expanded (PANAS-X), subscale sadness | PO | Some concerns |
| Beliefs about medicines predict side-effects of placebo modafinil | Heller et al., 2022 | United Kingdom | Cognitive performance | Non-clinical | 1 | OLP- pill | 65 (55.7) | 22.9 (5) | low | NT | 68 (55.7) | 22.9 (5) | Wechsler Auditory Digit Span Test (WDST)  Continuous Performance Test-AX version (CPT-AX) | Perceived cognitive enhancement, VAS | Outcomes related to hypothesis of the primary study | Some concerns |
| Open-label placebo treatment for cancer-related fatigue: A randomized-controlled clinical trial | Hoenemeyer et al., 2018 | USA | Cancer-related fatigue | Clinical | 21 | OLP pill | 38 (72) | 58.4 (11.2) | high | WL | 35 (66) | 56 (12.4) |  | Fatigue Symptom Inventory (FSI-14)  Multidimensional Fatigue Symptom Inventory Short Form (MFSI-SF30) | PO | Some concerns |
| Open-label placebo trial among japanese patients with chronic low back pain | Ikemoto et al., 2020 | Japan | Chronic low back pain | Clinical | 84 | OLP pill | 26 (65.4) | 68.2 (13.0) | high | TAU | 26 (57.7) | 65.3 (13.8) | Timed-Up-and-Go (TUG) in seconds | Roland–Morris Disability Questionnaire (RMDQ)  pain intensity, NRS | PO | High |
| Placebos without deception: A randomized controlled trial in irritable bowel syndrome | Kaptchuk et al., 2010 | USA | Irritable bowel syndrome | Clinical | 21 | OLP pill | 37 (65.0) | 47.0 (18.0) | high | NT | 43 (74.0) | 46.0 (18.0) |  | IBS Global Improvement Scale (IBS-GIS) | PO | Some concerns |
| Open-label placebo for major depressive disorder: A pilot randomized controlled trial | Kelley et al., 2012 | USA | Major depressive disorder | Clinical | 14 | OLP pill | 11 (70) | 38.8 (12.6) | high | WL | 9 (70) | 38.8 (12.6) | 17-item Hamilton Scale for Depression (HAM-D-17) |  | PO | High |
| Effects of open-label placebo on pain, functional disability, and spine mobility in patients with chronic back pain: A randomized controlled trial | Kleine-Borgmann et al., 2019 | Germany | Chronic low back pain | Clinical | 21 | OLP pill | 63 (55.6) | 60.3 (15.2) | high | WL | 59 (69.5) | 58.4 (14.0) |  | Composite pain intensity score, NRS | PO | Some concerns |
| Effects of open-label placebos on test performance and psychological well-being in healthy medical students: A randomized controlled trial | Kleine-Borgmann et al., 2021 | Germany | Cognitive performance | Non-clinical | 21 | OLP pill | 79 (68) | 24 (2.8) | high | NT | 75 (48.7) | 24 (2.7) | Result of midterm medical exam score |  | PO | Some concerns |
| Deceptive and nondeceptive placebos to reduce pain: An experimental study in healthy individuals | Kube et al., 2020 | Germany | Exp. pain | Non-clinical | 1 | OLP cream (expectancy & hope) | 62 (62) | 25.2 (6.4) | high | NT | 25 (38.5) | 24.9 (5.8) | Pain tolerance (°C) | Pain intensity, VAS  Pain unpleasantness, VAS | PO | Some concerns |
| Providing open-label placebos remotely - a randomized controlled trial in allergic rhinitis | Kube et al., 2021 | Germany | Allergic rhinitis | Clinical | 14 | OLP pill (augmented & limited) | 28 (64.3) | 27.0 (10.6) | high | WL (augmented & limited) | 26 (73.1) | 36 (14.8) |  | Combined Symptom Medication Score (CSMS) | PO | Some concerns |
| Can placebos reduce intrusive memories? | Kube et al., 2022a | Germany | Emotional distress – intrusive memories | Non-clinical | 7 | OLP pill | 36 (63.8) | 26.7 (7) | high | NT | 38 (68.4) | 25.5 (6.2) |  | Quantity of intrusive memories, NRS  Intensity of intrusive memories, 10-point Likert scale  Distress related to intrusive memories, 10-point Likert scale | POs | Some concerns |
| Remotely provided open-label placebo reduces frequency of and impairment by allergic symptoms | Kube et al., 2022b | Germany | Allergic rhinitis | Clinical | 14 | OLP pill | 35 (73) | 32.9 (12.5) | high | NT | 39 (65.8) | 32 (13.6) |  | Combined Symptom Medication Score (CSMS)  **C**omposite allergic symptoms score by Schäfer et al. 2016, 2018 | POs | Some concerns |
| Open-label placebo vs double-blind placebo for irritable bowel syndrome: A randomized clinical trial | Lembo et al., 2021 | USA | Irritable bowel syndrome | Clinical | 42 | OLP pill | 89 (71.9) | 42.2 (17.8) | high | NT | 86 (73.3) | 40 (17) |  | IBS severity score system (IBS-SSS) | PO | Some concerns |
| Is the rationale more important than deception? A randomized controlled trial of open-label placebo analgesia | Locher et al., 2017 | Switzerland | Exp. Pain | Non-clinical | 1 | OLP cream | 37 (73.0) | 25.7 (7.8) | high | NT | 40 (73) | 27.9 (8.5) | Pain tolerance(°C) | Pain intensity, VAS;  Pain unpleasantness; VAS | POs | Low |
|  |  |  |  |  |  | OLP cream | 37 (65.0) | 28.3 (11.3) | low |  |  |  |  |  |  |  |
| Open-label placebos for wound healing: A randomized controlled trial | Mathur et al., 2018 | New Zealand | Exp. wound healing | Non-clinical | 7 | OLP pill | 32 (87.9) | 28.8 (12.2) | high | NT | 33 (87.9) | 26.7 (8.4) | Area of the wound healed in percent (after 7 & 10 days) |  | POs | Low |
| Placebo effects of open-label verbal suggestions on itch | Meeuwis et al., 2018 | Netherlands | Exp. itch | Non-clinical | 1 | OLP verbal suggestion | 45 (81.5) | 21.3 (1.9) | high | NT | 46 (81.5) | 21.3 (1.9) |  | Itch, NRS | PO | Some concerns |
| Reduction in caffeine withdrawal after open-label decaffeinated coffee | Mills et al., 2023 | Australia | Caffeine withdrawal | Non-clinical | 1 | OLP- decaffeinated coffee | 22 (55) | 26.5 (10.6) | low | NT (water) | 20 (70) | 26.2 (8.8) |  | Caffeine Withdrawal Symptom Questionnaire (CWSQ) | PO | High |
| Conditioning open-label placebo: A pilot pharmacobehavioral approach for opioid dose  reduction and pain control | Morales-Quezada et al., 2020 | USA | Pain | Clinical | 6 | cOLP pill | 10 (30) | 44.9 (16.9) | high | TAU | 10 (30) | 49.7 (16.6) | MEDC (morphine equivalent dose conversion) |  | PO | Some concerns |
| A comparison of deceptive and non-deceptive placebo analgesia: Efficacy and ethical consequences | Mundt et al., 2017 | USA | Exp. Pain | Non-clinical | 1 | OLP cream | 25 (57.3) | 22.8 (5.9) | high | CP | 25 (57.3) | 22.8 (5.9) |  | Pain intensity, VAS | Outcome related to hypothesis of the primary study | Some concerns |
| Open-label placebo for the treatment of unipolar depression: Results from a randomized controlled trial. | Nitzan et al., 2020 | Israel | Major depressive disorder | Clinical | 28 | OLP pill | 18 (77.8) | 48.2 (16.9) | high | WL | 20 (70) | 51.7 (17.7) |  | Quick inventory of Depressive Symptomatology (QIDS) | PO | Some concerns |
| Effect of open-label placebo on children and adolescents with functional abdominal pain or irritable bowel syndrome | Nurko et al., 2022 | USA | Functional abdominal pain or irritable bowel syndrome | Clinical | 21 | OLP syrup | 15 (80.0) | 13.8 (3.2) | high | WL | 15 (80.0) | 14.4 (3.5) |  | Pain intensity, VAS | PO before cross-over | Some concerns |
| Open-label placebo administration decreases pain in elderly patients with symptomatic knee osteoarthritis – a randomized controlled trial | Olliges et al., 2022 | Germany | Knee osteoarthritis - pain | Clinical | 21 | OLP pills (pain & mood) | 41 (77.4) | 65.8 (9.5) | high | NT | 19 (63.2) | 69.9 (9.6) |  | Knee pain, NRS  Western Ontario and McMaster Universities Osteoarthritis Index Questionnaire (WOMAC) | POs in continuous metric | Some concerns |
| Effects of (non)deceptive placebos on reported sleep quality and food cue reactivity | Potthoff & Schienle, 2023 | Austria | Sleep quality | Non-clinical | 7 | OLP oral spray | 36 (71.8) | 31 (14) | high | NT | 43 (69.6) | 27.9 (10.6) |  | Pittsburgh Sleep Quality Index (PSQI)), subscale 1-7 | Outcomes related to OLP intervention | High |
| Open-label placebo interventions with drinking water and their influence on perceived physical and mental well-being | Rathschlag & Klatt, 2021a | Germany | Physical and Mental Well-Being | Non-clinical | 14 | OLP pill | 18 (27.9) | 22 (3.7) | high | NT | 16 (27.9) | 22 (3.7) |  | Acute Recovery and Stress Scale (ARSS)  Questionnaire for Assessing Subjective Physical Well Being (FEW-16) | All | Some concerns |
| Open-label placebo interventions with drinking water and their influence on perceived physical and mental well-being. | Rathschlag & Klatt, 2021b | Germany | Physical and Mental Well-Being | Non-clinical | 14 | OLP pill | 18 (60) | 26.5 (9.9) | high | NT | 19 (60) | 26.5 (9.9) |  | Acute Recovery and Stress Scale (ARSS)  Questionnaire for Assessing Subjective Physical Well Being (FEW-16) | All | Some concerns |
| The hidden effects of blinded, placebo-controlled randomized trials: An experimental investigation | Rief & Glombiewski, 2012 | Germany | Exp. Pain | Non-clinical | 1 | OLP nasal (active & passive) | 41 (65) | 24 (NA) | high | NT | 20 (55) | 23.2 (2.9) | Pain threshold (°C) |  | PO | Some concerns |
| Open-label placebos improve symptoms in allergic rhinitis: A randomized controlled trial | Schaefer et al., 2016 | Germany | Allergic rhinitis | Clinical | 14 | OLP pill | 11 (84) | 25 (9.9) | high | NT | 14 (84) | 23 (9.9) |  | Self-developed composite allergic symptoms score, 7-point Likert skale | Available PO | High |
| Why do open-label placebos work? A randomized controlled trial of an open-label placebo induction with and without extended information about the placebo effect in allergic rhinitis | Schaefer et al., 2018 | Germany | Allergic rhinitis | Clinical | 14 | OLP  pill | 13 (69.2) | 23.0 (3) | high | NT (with & without rationale) | 20 (95) | 13 (7.1) |  | Self-developed composite allergic symptoms score, 7-point Likert skale | PO | Some concerns |
|  |  |  |  |  |  | OLP- pill | 13 (69.2) | 25.0 (9) | low |  |  |  |  |  |  |  |
| Open-label placebos reduce test anxiety and improve self-management skills: A randomized-controlled trial | Schaefer et al., 2019 | Germany | Test anxiety | Non-clinical | 14 | OLP pill | 31 (80.6) | 22.3 (2.3) | high | NT | 27 (92.6) | 23.5 (2.3) |  | Brief German test anxiety inventory (PAF)  Questionnaire for Measuring Resources and Self-Manage ment Skills (FERUS) | All outcomes | Some concerns |
| Effects of open-label placebos on state anxiety and glucocorticoid stress responses | Schaefer et al., 2021 | Germany | Experimental acute stress | Non-clinical | 21 | OLP pill | 24 (58.3) | 25.3 (7.3) | high | NT | 29 (48.3) | 27.4 (10.3) |  | State dimension of the State Trait-Anxiety Inventory (STAI-S)  Positive and Negative Affect Scale (PANAS)  Current stress (VAS) | Self-reported outcomes | Some concerns |
| A randomized controlled trial of effects of open-label placebo compared to double-blind placebo and treatment-as-usual on symptoms of allergic rhinitis | Schaefer et al., 2023a | Germany | Allergic rhinitis | Clinical | 28 | OLP drops | 22 (45.5) | 28.1 (11) | high | NT | 22 (50) | 25.5 (6.8) |  | Combined Symptoms and Medication Score (CSMS), last 12h  Combined Symptoms and Medication Score (CSMS), last 2 weeks | POs | Some concerns |
| Neural underpinnings of open-label placebo effects in emotional distress | Schaefer et al., 2023b | Germany | Emotional distress - negative pictures | Non-clinical | 1 | OLP nasal spray | 28 (59.8) | 23.4 (7.3) | high | CP | 58 (59.8) | 23.4 (7.3) |  | Emotional distress on a nine-point Likert scale | Self-reported outcomes | Some concerns |
| Neural underpinnings of open-label placebo effects in emotional distress | Schaefer et al., 2023c | Germany | Emotional distress - negative pictures | Non-clinical | 1 | OLP nasal spray | 23 (100) | 22.5 (3) | high | CP | 21 (100) | 22.2 (2) |  | One-item rating scale: intensity of negative feelings | Self-reported outcomes | Some concerns |
| Open-label placebos as adjunctive therapy for patients with depression | Schienle & Jurinec, 2022 | Austria/ Slovenia | Major depressive disorder | Clinical | 28 | OLP drops | 30 (83) | 46 (11.7) | high | TAU | 30 (83) | 48.9 (11.8) |  | The Beck Depression Scale (BDI-II) | PO | Some concerns |
| Open-label placebo treatment to improve relaxation training effects in healthy psychology students: A randomized controlled trial | Schienle & Unger, 2021 | Austria | Promote behavior - Relaxation training | Non-clinical | 14 | OLP drops | 68 (71) | 24.4 (2.7) | high | TAU | 80 (71) | 24.4 (2.7) |  | Quantity of progressive muscle relaxation (PMR) exercises per week | Outcomes related to OLP intervention | High |
| Non-deceptive placebos can promote Acts of Kindness: A randomized controlled trial | Schienle & Unger, 2023 | Austria | Promote behavior - Act of kindness | Non-clinical | 7 | OLP drops | 71 (83.3) | 24.7 (3.8) | high | NT | 89 (83.3) | 24.7 (3.8) |  | Quantity of act of kindness (AoK) per week | Outcomes related to OLP intervention | Some concerns |
| Changes in neural processing and evaluation of negative facial expressions after administration of an open-label placebo | Schienle et al., 2022 | Austria | Emotional distress – negative pictures | Non-clinical | 1 | OLP nasal spray | 52 (100) | 22.5 (3.0) | high | CP | 51 (100) | 22.5 (3.7) | Early and late LPP | Arousal and perceived intensity of anger, VAS | Preregistered outcomes | Some concerns |
| A randomized trial that compared brain activity, efficacy and plausibility of open-label placebo treatment and cognitive repappraisal for reducing emotional distress | Schienle et al., 2023 | Austria | Emotional distress – negative pictures | Non-clinical | 1 | OLP pill | 48 (100) | 23.5 (5.2) | high | CP | 49 (100) | 23.5 (5.2) |  | Disgust intensity scale, 9-point Likert scale | Self-report outcomes with pre to post scores | Some concerns |
| Effects of expectation and caffeine on arousal, well-being, and reaction time | Schneider et al., 2006 | Germany | Arousal, well-being, | Non-clinical | 1 | OLP- decaffeinated coffee | 15 (77.8) | 31.0 (11.8) | low | NT | 15 (77.8) | 31.0 (11.8) | Systolic blood pressure (mmHg)  Diastolic blood pressure (mmHg)  Heart rate (bpm) Reaction time (ms) | Multi-dimensional Well Being Questionnaire (all 3 dimensions) | All | Some concerns |
| Deceptive and open-label placebo effects in experimentally induced guilt: A randomized controlled trial in healthy subjects | Sezer et al., 2022 | Switzerland | Emotional distress – exp. guilt | Non-clinical | 1 | OLP pill | 35 (74.3) | 24 (5.6) | high | NT | 39 (64.1) | 21.7 (3.1) |  | State Shame and Guilt Scale (SSGS), guilt subscale | PO | Some concerns |
| Effects of an open-label placebo intervention on reactions to social exclusion in healthy adults: A randomized controlled trial | Stumpp et al., 2023 | Switzerland | Emotional distress – exp. social exclusion | Non-clinical | 1 | OLP pill | 21 (71.3) | 23.7 (6.6) | high | NT | 15 (73.3) | 29.5 (7.4) |  | Need threat scale, 9-point semantic differential scale  Hurt feelings on a 9-point Likert-type item | Preregistered outcomes | Some concerns |
| Effect of acupuncture and instruction on physiological recovery from maximal exercise: A balanced-placebo controlled trial | Urroz et al., 2016 | Australia | Physiological recovery | Non-clinical | 1 | OLP- sham acupuncture | 12 (41.7) | 21.3 (1.5) | low | NT | 12 (33.3) | 21.8 (1.9) | Systolic blood pressure (mmHg)  Diastolic blood pressure (mmHg)  Heart rate (bpm)  Volume of oxy gen consump tion (ml/min)  Respiratory rate (breaths/min)  Blood lactate (mmol/l) |  | All | Some concerns |
| The effects of a caffeine placebo and experimenter expectation on blood pressure, heart rate, well-being, and cognitive performance | Walach et al., 2001 | Germany | Arousal, well-being | Non-clinical | 1 | OLP decaffeinated coffe | 41 (66.2) | 28.1 (7.6) | high | NT | 37 (66.2) | 28.1 (7.6) | Systolic blood pressure (mmHg) Diastolic blood pressure (mmHg) Heart rate (bpm) | Basle Well Being Scale | All | Low |
| The impact of pharmaceutical form and simulated side effects in an open-label-placebo RCT for improving psychological distress in highly stressed students | Winkler et al., 2023 | Germany | Acute Stress | non-Nclinical | 7 | OLP pill & nasal spray (active & passive) | 133 (81.2) | 23.7 (3) | high | NT | 45 (82.2) | 23.5 (3.5) |  | Perceived Stress Scale-10 (PSS-10)  Depression Anxiety and Stress Scale 21 (DASS-21) | POs | High |
| Open-label placebo for the treatment of cancer-related fatigue in patients with advanced cancer: A randomized controlled trial | Yennurajalingam et al., 2022 | USA | Cancer-related fatigue | Clinical | 7 | OLP pill | 42 (74) | 57 (12) | high | WL | 42 (60) | 55 (14) |  | Functional Assessment of Chronic Illness Therapy-Fatigue (FACIT-F) | PO | High |
| Open-label placebo reduces fatigue in cancer survivors: A randomized trial | Zhou et al., 2019 | USA | Cancer-related fatigue | Clinical | 22 | OLP pill | 20 (92.5) | 47.3 (12.4) | high | WL | 20 (92.5) | 47.3 (12.4) |  | Functional Assessment of Chronic Illness Therapy-Fatigue (FACIT-F) | PO | Some concerns |

*Note*. ^a^ OLP = open-label placebo. ^b^ N = number of participants. ^c^ IG = intervention group. ^d^ SD = standard deviation. ^e^ CG = control group. ^f^ NT = no treatment. ^g^ NRS = numeric rating scale. ^h^ PO = primary outcome. ^i^ Exp. = experimental. ^j^ VAS = visual analog scale. ^k^ OLP- = open-label-placebo without suggestive treatment rationale. ^l^ cOLP = conditioned open-label placebo. ^m^ TAU = treatment as usual. ^n^ WL = waiting list. ^o^ CP = covert placebo.

## Supplemental digital appendix A7: Numerical characteristics of included trials

| Authors, Year | Form of report | Measure | Type score | Mean IG^a^ pre (SD)^b^ | N^c^ IG pre | Mean CG^d^ pre (SD) | N CG pre | Mean IG post (SD) | N IGpost | Mean CG post (SD) | N CG post | Change IG provided (SD) | N change IG provided | Change CG provided (SD) | N change CG provided |
| --- | --- | --- | --- | --- | --- | --- | --- | --- | --- | --- | --- | --- | --- | --- | --- |
| Ashar et al., 2022 | Self-report | Average pain last week | Change | -4.16 (1.33) | 51.00 | -3.91 (1.34) | 50.00 | -2.84 (1.64) | 44.00 | -3.13 (1.45) | 47.00 | n.ae (n.a.) | n.a. | n.a. (n.a.) | n.a. |
| Barnes et al., 2019a | Self-report | Nausea OLP fully & semi vs CG | Change | n.a. (n.a.) | n.a. | n.a. (n.a.) | n.a. | n.a. (n.a.) | n.a. | n.a. (n.a.) | n.a. | 6.28 (6.54) | 29.00 | 7.83 (6.68) | 15.00 |
| Barnes et al., 2019b | Self-report | Nausea OLP semi vs CG | Change | n.a. (n.a.) | n.a. | n.a. (n.a.) | n.a. | n.a. (n.a.) | n.a. | n.a. (n.a.) | n.a. | 21.52 (14.06) | 31.00 | 22.42 (13.91) | 31.00 |
| Barnes et al., 2023 -^f^ | Self-report | Negative emotions OLP-^f^ | Change | -11.10 (7.20) | 35.00 | -12.50 (9.30) | 32.00 | -9.70 (9.00) | 35.00 | -12.50 (10.80) | 32.00 | n.a. (n.a.) | n.a. | n.a. (n.a.) | n.a. |
| Barnes et al., 2023 - | Self-report | Physical symptoms OLP- | Change | -12.50 (9.80) | 35.00 | -14.40 (10.00) | 32.00 |  | 35.00 |  | 32.00 |  | n.a. |  | n.a. |
| Barnes et al., 2023 - | Self-report | Positive mental well-being OLP- | Change | 46.60 (7.80) | 35.00 | 48.30 (7.50) | 32.00 | 46.10 (9.00) | 35.00 | 46.00 (10.40) | 32.00 | n.a. (n.a.) | n.a. | n.a. (n.a.) | n.a. |
| Barnes et al., 2023 - | Self-report | Sleep quality OLP- | Change | -9.70 (5.60) | 35.00 | -9.30 (5.30) | 32.00 | -8.30 (5.50) | 35.00 | -9.70 (5.90) | 32.00 | n.a. (n.a.) | n.a. | n.a. (n.a.) | n.a. |
| Barnes et al., 2023 +^g^ | Self-report | Negative emotions OLP+^g^ | Change | -13.90 (11.50) | 35.00 | -12.50 (9.30) | 32.00 | -7.90 (7.30) | 35.00 | -12.50 (10.80) | 32.00 | n.a. (n.a.) | n.a. | n.a. (n.a.) | n.a. |
| Barnes et al., 2023 + | Self-report | Physical symptoms OLP+ | Change | -16.20 (11.70) | 35.00 | -14.40 (10.00) | 32.00 | -10.70 (9.10) | 35.00 | -13.90 (11.20) | 32.00 | n.a. (n.a.) | n.a. | n.a. (n.a.) | n.a. |
| Barnes et al., 2023 + | Self-report | Positive mental well-being OLP+ | Change | 47.50 (8.40) | 35.00 | 48.30 (7.50) | 32.00 | 49.20 (8.60) | 35.00 | 46.00 (10.40) | 32.00 | n.a. (n.a.) | n.a. | n.a. (n.a.) | n.a. |
| Barnes et al., 2023 + | Self-report | Sleep quality OLP+ | Change | -9.90 (5.20) | 35.00 | -9.30 (5.30) | 32.00 | -7.30 (4.60) | 35.00 | -9.70 (5.90) | 32.00 | n.a. (n.a.) | n.a. | n.a. (n.a.) | n.a. |
| Belcher et al., 2023 | Objective | Methadone dose | Change | -26.10 (4.19) | 77.00 | -27.06 (3.40) | 54.00 | -79.40 (19.60) | 60.00 | -83.10 (25.10) | 33.00 | n.a. (n.a.) | n.a. | n.a. (n.a.) | n.a. |
| Buergler et al., 2023 | Self-report | Test anxiety OLP+IP | Change | -50.67 (9.32) | 114.00 | -50.66 (7.37) | 59.00 | -46.19 (9.62) | 114.00 | -52.10 (9.56) | 59.00 | n.a. (n.a.) | n.a. | n.a. (n.a.) | n.a. |
| Carvalho et al., 2016 | Self-report | Dysfunction | Change | n.a. (n.a.) | n.a. | n.a. (n.a.) | n.a. | n.a. (n.a.) | n.a. | n.a. (n.a.) | n.a. | 2.86 (3.91) | 41.00 | 0.02 (3.73) | 42.00 |
| Carvalho et al., 2016 | Self-report | Low back pain | Change | n.a. (n.a.) | n.a. | n.a. (n.a.) | n.a. | n.a. (n.a.) | n.a. | n.a. (n.a.) | n.a. | 1.49 (1.68) | 41.00 | 0.24 (1.61) | 42.00 |
| El Brihi et al., 2019 | Self-report | Emotional distress | Change | n.a. (n.a.) | n.a. | n.a. (n.a.) | n.a. | n.a. (n.a.) | n.a. | n.a. (n.a.) | n.a. | 7.28 (8.81) | 61.00 | 0.19 (6.42) | 27.00 |
| El Brihi et al., 2019 | Self-report | Physical symptoms | Change | n.a. (n.a.) | n.a. | n.a. (n.a.) | n.a. | n.a. (n.a.) | n.a. | n.a. (n.a.) | n.a. | 8.65 (9.02) | 61.00 | 0.81 (19.05) | 26.00 |
| El Brihi et al., 2019 | Self-report | Positive mental well-being | Change | n.a. (n.a.) | n.a. | n.a. (n.a.) | n.a. | n.a. (n.a.) | n.a. | n.a. (n.a.) | n.a. | 3.57 (8.94) | 61.00 | -2.30 (6.27) | 27.00 |
| El Brihi et al., 2019 | Self-report | Sleep quality | Change | n.a. (n.a.) | n.a. | n.a. (n.a.) | n.a. | n.a. (n.a.) | n.a. | n.a. (n.a.) | n.a. | 1.70 (2.06) | 61.00 | 0.17 (1.42) | 26.00 |
| Flowers et al. 2021 | Self-report | Average pain last 24h | Change | -5.35 (2.56) | 26 | -5.36 (1.37) | 25 | -3.22 (1.51) | 16 | -3,98 (2.32) | 19 | n.a. (n.a.) | n.a. | n.a. (n.a.) | n.a. |
| Flowers et al. 2021 | Objective | Morphine milligram equivalents | Post-only | n.a. (n.a.) | n.a. | n.a. (n.a.) | n.a. | -9.11 (17.6) | 16 | -22.6 (29.5) | 18 | n.a. (n.a.) | n.a. | n.a. (n.a.) | n.a. |
| Friehs et al., 2022 | Self-report | Sadness | Change | -6.10 (8.70) | 63.00 | -3.30 (5.30) | 29.00 | -11.20 (9.50) | 63.00 | -11.50 (9.00) | 29.00 | n.a. (n.a.) | n.a. | n.a. (n.a.) | n.a. |
| Glombiewski et al. 2019 | Self-report | Sadness OLP | Change | -10.96 (12.95) | 32.00 | -10.26 (9.27) | 32.00 | -16.99 (13.02) | 32.00 | -19.67 (11.43) | 32.00 | n.a. (n.a.) | n.a. | n.a. (n.a.) | n.a. |
| Guevarra et al., 2020a | Self-report | Self-reported emotional distress | Post-only | n.a. (n.a.) | n.a. | n.a. (n.a.) | n.a. | n.a. (n.a.) | n.a. | n.a. (n.a.) | n.a. | -6.19 (1.48) | 29.00 | -7.50 (1.13) | 33.00 |
| Guevarra et al., 2020b | Objective | Emotional distress (sustained LPP, 1000–2000 ms) | Post-only | n.a. (n.a.) | n.a. | n.a. (n.a.) | n.a. | n.a. (n.a.) | n.a. | n.a. (n.a.) | n.a. | -1.79 (3.88) | 99.00 | -2.59 (3.88) | 99.00 |
| Guevarra et al., 2020b | Objective | Emotional distress (sustained LPP, 2000–3000 ms) | Post-only | n.a. (n.a.) | n.a. | n.a. (n.a.) | n.a. | n.a. (n.a.) | n.a. | n.a. (n.a.) | n.a. | -1.09 (5.07) | 99.00 | -2.84 (5.07) | 99.00 |
| Guevarra et al., 2020b | Objective | Emotional distress (sustained LPP, 3000–4000 ms) | Post-only | n.a. (n.a.) | n.a. | n.a. (n.a.) | n.a. | n.a. (n.a.) | n.a. | n.a. (n.a.) | n.a. | -0.54 (5.37) | 99.00 | -2.96 (5.37) | 99.00 |
| Guevarra et al., 2020b | Objective | Emotional distress (sustained LPP, 4000–5000 ms) | Post-only | n.a. (n.a.) | n.a. | n.a. (n.a.) | n.a. | n.a. (n.a.) | n.a. | n.a. (n.a.) | n.a. | -0.36 (5.67) | 99.00 | -2.93 (5.67) | 99.00 |
| Guevarra et al., 2020b | Objective | Emotional distress (sustained LPP, 5000–6000 ms) | Post-only | n.a. (n.a.) | n.a. | n.a. (n.a.) | n.a. | n.a. (n.a.) | n.a. | n.a. (n.a.) | n.a. | -0.50 (5.87) | 99.00 | -3.02 (5.87) | 99.00 |
| Haas et al., 2020 | Self-report | Sadness | Change | -60.08 (20.23) | 24.00 | -46.16 (20.26) | 22.00 | -58.25 (20.58) | 24.00 | -52.31 (20.64) | 22.00 | n.a. (n.a.) | n.a. | n.a. (n.a.) | n.a. |
| Hahn et al., 2022 | Self-report | Sadness | Change |  | 42.00 |  | 42.00 |  | 42.00 |  | 42.00 |  | n.a. |  | n.a. |
| Heller et al., 2022 | Objective | Backward digit span | Post-only | n.a. (n.a.) | n.a. | n.a. (n.a.) | n.a. | n.a. (n.a.) | n.a. | n.a. (n.a.) | n.a. | 9.57 (2.59) | 65.00 | 10.35 (2.15) | 68.00 |
| Heller et al., 2022 | Objective | Correct target responses | Post-only | n.a. (n.a.) | n.a. | n.a. (n.a.) | n.a. | n.a. (n.a.) | n.a. | n.a. (n.a.) | n.a. | 24.46 (5.98) | 65.00 | 25.54 (3.10) | 68.00 |
| Heller et al., 2022 | Objective | Forward digit span | Post-only | n.a. (n.a.) | n.a. | n.a. (n.a.) | n.a. | n.a. (n.a.) | n.a. | n.a. (n.a.) | n.a. | 9.97 (2.42) | 65.00 | 10.09 (2.33) | 68.00 |
| Heller et al., 2022 | Objective | Reaction time in ms | Post-only | n.a. (n.a.) | n.a. | n.a. (n.a.) | n.a. | n.a. (n.a.) | n.a. | n.a. (n.a.) | n.a. | -195.96 (98.99) | 65.00 | -171.61 (64.33) | 68.00 |
| Heller et al., 2022 | Self-report | Perceived cognitive enhancement | Post-only | n.a. (n.a.) | n.a. | n.a. (n.a.) | n.a. | n.a. (n.a.) | n.a. | n.a. (n.a.) | n.a. | 53.38 (12.22) | 65.00 | 57.53 (14.73) | 68.00 |
| Hoenemeyer et al., 2018 | Self-report | Cancer related fatigue | Change | -64.30 (23.30) | 38.00 | -59.00 (21.10) | 35.00 | -45.70 (22.70) | 38.00 | -52.90 (24.10) | 35.00 | n.a. (n.a.) | n.a. | n.a. (n.a.) | n.a. |
| Hoenemeyer et al., 2018 | Self-report | Cancer related fatigue | Change | -33.90 (17.80) | 38.00 | -27.40 (19.30) | 35.00 | -20.80 (19.50) | 38.00 | -26.00 (21.50) | 35.00 | n.a. (n.a.) | n.a. | n.a. (n.a.) | n.a. |
| Ikemoto et al., 2020 | Objective | Basic mobility | Change | n.a. (n.a.) | n.a. | n.a. (n.a.) | n.a. | n.a. (n.a.) | n.a. | n.a. (n.a.) | n.a. | 0.60 (1.50) | 24.00 | 1.10 (1.10) | 24.00 |
| Ikemoto et al., 2020 | Self-report | Pain severity | Change | n.a. (n.a.) | n.a. | n.a. (n.a.) | n.a. | n.a. (n.a.) | n.a. | n.a. (n.a.) | n.a. | 1.10 (1.90) | 24.00 | 0.80 (1.90) | 24.00 |
| Ikemoto et al., 2020 | Self-report | Subjective disability | Change | n.a. (n.a.) | n.a. | n.a. (n.a.) | n.a. | n.a. (n.a.) | n.a. | n.a. (n.a.) | n.a. | 3.30 (3.20) | 24.00 | 2.30 (3.20) | 24.00 |
| Kaptchuk et al., 2010 | Self-report | Irritable bowl symptoms | Post-only | n.a. (n.a.) | n.a. | n.a. (n.a.) | n.a. | n.a. (n.a.) | n.a. | n.a. (n.a.) | n.a. | 5.00 (1.50) | 37.00 | 3,90 (1.30) | 43.00 |
| Kelley et al., 2012 | Objective | Depression | Change | n.a. (n.a.) | n.a. | n.a. (n.a.) | n.a. | n.a. (n.a.) | n.a. | n.a. (n.a.) | n.a. | 1.64 (4.52) | 11.00 | 0.67 (4.00) | 9.00 |
| Kleine-Borgmann et al., 2019 | Self-report | Pain | Change | n.a. (n.a.) | n.a. | n.a. (n.a.) | n.a. | n.a. (n.a.) | n.a. | n.a. (n.a.) | n.a. | 0.62 (1.83) | 63.00 | 0.11 (1.31) | 59.00 |
| Kleine-Borgmann et al., 2021 | Objective | Test performance | Post-only | n.a. (n.a.) | n.a. | n.a. (n.a.) | n.a. |  | n.a. |  | n.a. |  | 79.00 |  | 75.00 |
| Kube et al., 2020 | Objective | Heat pain tolerance OLP-H+OLP-E | Change | 48.26 (1.45) | 50.00 | 48.52 (1.33) | 25.00 | 48.48 (1.47) | 50.00 | 48.44 (1.26) | 25.00 | n.a. (n.a.) | n.a. | n.a. (n.a.) | n.a. |
| Kube et al., 2020 | Self-report | Heat pain intensity OLP-H+OLP-E | Change | -82.61 (13.82) | 50.00 | -77.42 (14.07) | 25.00 | -82.63 (13.44) | 50.00 | -80.12 (13.76) | 25.00 | n.a. (n.a.) | n.a. | n.a. (n.a.) | n.a. |
| Kube et al., 2020 | Self-report | Heat pain unpleasentness OLP-H+OLP-E | Change | -80.92 (14.66) | 50.00 | -70.08 (20.08) | 25.00 | -80.63 (14.96) | 50.00 | -70.65 (18.69) | 25.00 | n.a. (n.a.) | n.a. | n.a. (n.a.) | n.a. |
| Kube et al., 2021 | Self-report | Allergic symptoms | Change | -16.20 (2.90) | 14.00 | -15.90 (4.30) | 13.00 | -14.90 (3.80) | 14.00 | -12.10 (3.30) | 13.00 | n.a. (n.a.) | n.a. | n.a. (n.a.) | n.a. |
| Kube et al., 2021 | Self-report | Allergic symptoms | Change | -15.10 (4.80) | 14.00 | -15.60 (4.80) | 13.00 | -12.00 (3.00) | 14.00 | -13.60 (3.50) | 13.00 | n.a. (n.a.) | n.a. | n.a. (n.a.) | n.a. |
| Kube et al., 2022a | Self-report | Distress | Post-only | n.a. (n.a.) | n.a. | n.a. (n.a.) | n.a. | n.a. (n.a.) | n.a. | n.a. (n.a.) | n.a. | -2.84 (0.98) | 36.00 | -3.53 (1.34) | 38.00 |
| Kube et al., 2022a | Self-report | Intrusion frequency | Post-only | n.a. (n.a.) | n.a. | n.a. (n.a.) | n.a. | n.a. (n.a.) | n.a. | n.a. (n.a.) | n.a. | -3.61 (3.75) | 36.00 | -3.87 (3.34) | 38.00 |
| Kube et al., 2022a | Self-report | Intrusion intensity | Post-only | n.a. (n.a.) | n.a. | n.a. (n.a.) | n.a. | n.a. (n.a.) | n.a. | n.a. (n.a.) | n.a. | -3.63 (1.22) | 36.00 | -4.26 (1.72) | 38.00 |
| Kube et al., 2022b | Self-report | Allergic symptoms:frequency | Change | -2.92 (0.82) | 35.00 | -2.73 (0.84) | 39.00 | -1.94 (0.73) | 35.00 | -2.25 (0.70) | 39.00 | n.a. (n.a.) | n.a. | n.a. (n.a.) | n.a. |
| Kube et al., 2022b | Self-report | Allergic symptoms:severity | Change | -1.58 (0.55) | 35.00 | -1.62 (0.76) | 39.00 | -0.93 (0.61) | 35.00 | -1.20 (0.66) | 39.00 | n.a. (n.a.) | n.a. | n.a. (n.a.) | n.a. |
| Lembo et al., 2021 | Self-report | Irritable bowl symptoms | Change | n.a. (n.a.) | n.a. | n.a. (n.a.) | n.a. | n.a. (n.a.) | n.a. | n.a. (n.a.) | n.a. | 90.60 (89.50) | 68.00 | 52.30 (87.00) | 72.00 |
| Locher et al., 2017 - | Objective | Heat pain tolerance OLP- | Change | n.a. (n.a.) | n.a. | n.a. (n.a.) | n.a. | n.a. (n.a.) | n.a. | n.a. (n.a.) | n.a. | 47.99 (0.14) | 37.00 | 48.07 (0.14) | 40.00 |
| Locher et al., 2017 - | Self-report | Intensity OLP- | Change | n.a. (n.a.) | n.a. | n.a. (n.a.) | n.a. | n.a. (n.a.) | n.a. | n.a. (n.a.) | n.a. | -62.13 (1.42) | 37.00 | -60.35 (1.36) | 40.00 |
| Locher et al., 2017 - | Self-report | Unpleasantness OLP- | Change | n.a. (n.a.) | n.a. | n.a. (n.a.) | n.a. | n.a. (n.a.) | n.a. | n.a. (n.a.) | n.a. | -64.47 (1.54) | 37.00 | -63.80 (1.48) | 40.00 |
| Locher et al., 2017 + | Objective | heat pain toleranceOLP+ | Change | n.a. (n.a.) | n.a. | n.a. (n.a.) | n.a. | n.a. (n.a.) | n.a. | n.a. (n.a.) | n.a. | 48.15 (0.14) | 37.00 | 48.07 (0.14) | 40.00 |
| Locher et al., 2017 + | Self-report | Intensity OLP+ | Change | n.a. (n.a.) | n.a. | n.a. (n.a.) | n.a. | n.a. (n.a.) | n.a. | n.a. (n.a.) | n.a. | -59.51 (1.42) | 37.00 | -60.35 (1.36) | 40.00 |
| Locher et al., 2017 + | Self-report | Unpleasantness OLP+ | Change | n.a. (n.a.) | n.a. | n.a. (n.a.) | n.a. | n.a. (n.a.) | n.a. | n.a. (n.a.) | n.a. | -59.93 (1.54) | 37.00 | -63.80 (1.48) | 40.00 |
| Mathur et al., 2018 | Objective | After 10 days | Change | n.a. (n.a.) | n.a. | n.a. (n.a.) | n.a. | n.a. (n.a.) | n.a. | n.a. (n.a.) | n.a. | 0.93 (0.11) | 32.00 | 0.97 (0.06) | 33.00 |
| Mathur et al., 2018 | Objective | After 7 days | Change | n.a. (n.a.) | n.a. | n.a. (n.a.) | n.a. | n.a. (n.a.) | n.a. | n.a. (n.a.) | n.a. | 0.68 (0.32) | 32.00 | 0.73 (0.29) | 33.00 |
| Meeuwis et al., 2018 | Self-report | Histamine iontophoreisis | Change | -0.47 (0.84) | 45.00 | -0.39 (0.89) | 46.00 | -3.14 (1.61) | 45.00 | -3.46 (1.51) | 46.00 | n.a. (n.a.) | n.a. | n.a. (n.a.) | n.a. |
| Mills et al., 2023 | Self-report | Caffeine Withdrawal Symptom | Change | n.a. (n.a.) | n.a. | n.a. (n.a.) | n.a. | n.a. (n.a.) | n.a. | n.a. (n.a.) | n.a. | 9.50 (10.00) | 22.00 | 0.60 (5.00) | 19.00 |
| Morales-Quezada et al., 2020 | Objective | MEDC (morphine equivalent dose) | Change | -122.25 (114.90) | 10 | -65.63 (53.82) | 10 | -56.25 (60.51) | 10 | -61.87 (58.87) | 10 | n.a. (n.a.) | n.a. | n.a. (n.a.) | n.a. |
| Mundt et al., 2017 | Self-report | Pain intensity | Change | -41.90 (7.22) | 25.00 | -42.79 (10.69) | 25.00 | -41.69 (13.43) | 25.00 | -48.93 (11.87) | 25.00 | n.a. (n.a.) | n.a. | n.a. (n.a.) | n.a. |
| Nurko et al., 2022 | Self-report | Pain intensity | Change | -44.1 (16.0) | 15 | -47.5 (15.1) | 15 | -42.9 (14.0) | 15 | -45.4 (13.9) | 15 | n.a. (n.a.) | n.a. | n.a. (n.a.) | n.a. |
| Nitzan et al., 2020 | Self-report | Depression | Change | -11.28 (5.14) | 18.00 | -11.60 (4.47) | 20.00 | -9.33 (4.97) | 18.00 | -11.15 (3.65) | 20.00 | n.a. (n.a.) | n.a. | n.a. (n.a.) | n.a. |
| Olliges et al., 2022 | Self-report | Osteoarthritis: pain intensity OLP-pain+OLP-mood | Change | -23.07 (7.79) | 41.00 | -21.37 (8.02) | 19.00 | -18.17 (8.16) | 41.00 | -20.68 (8.76) | 19.00 | n.a. (n.a.) | n.a. | n.a. (n.a.) | n.a. |
| Olliges et al., 2022 | Self-report | Osteoarthritis: physical functioning OLP-pain+OLP-mood | Change | -76.04 (19.89) | 41.00 | -72.79 (32.03) | 19.00 | -62.05 (27.39) | 41.00 | -67.47 (30.20) | 19.00 | n.a. (n.a.) | n.a. | n.a. (n.a.) | n.a. |
| Olliges et al., 2022 | Self-report | Osteoarthritis: stiffness OLP-pain+OLP-mood | Change | -4.71 (5.82) | 41.00 | -10.74 (4.69) | 19.00 | -8.15 (3.69) | 41.00 | -9.63 (4.47) | 19.00 | n.a. (n.a.) | n.a. | n.a. (n.a.) | n.a. |
| Olliges et al., 2022 | Self-report | Pain intensity OLP-pain+OLP-mood | Change | -2.51 (1.48) | 41.00 | -2.63 (1.97) | 19.00 | -2.10 (1.59) | 41.00 | -2.91 (2.00) | 19.00 | n.a. (n.a.) | n.a. | n.a. (n.a.) | n.a. |
| Potthoff & Schienle, 2023 | Self-report | Daytime disfunction | Change | -1.03 (0.71) | 36.00 | -0.96 (0.84) | 43.00 | -0.94 (0.79) | 36.00 | -0.93 (0.74) | 43.00 | n.a. (n.a.) | n.a. | n.a. (n.a.) | n.a. |
| Potthoff & Schienle, 2023 | Self-report | Habitual sleep efficiency | Change | -0.36 (0.71) | 36.00 | -0.52 (0.94) | 43.00 | -0.64 (0.93) | 36.00 | -0.42 (0.82) | 43.00 | n.a. (n.a.) | n.a. | n.a. (n.a.) | n.a. |
| Potthoff & Schienle, 2023 | Self-report | Sleep disturbances | Change | -1.05 (0.51) | 36.00 | -1.04 (0.51) | 43.00 | -0.89 (0.58) | 36.00 | -1.05 (0.49) | 43.00 | n.a. (n.a.) | n.a. | n.a. (n.a.) | n.a. |
| Potthoff & Schienle, 2023 | Self-report | Sleep duration | Change | -0.21 (0.52) | 36.00 | -0.22 (0.42) | 43.00 | -0.39 (0.65) | 36.00 | -0.16 (0.43) | 43.00 | n.a. (n.a.) | n.a. | n.a. (n.a.) | n.a. |
| Potthoff & Schienle, 2023 | Self-report | Sleep latency | Change | -0.90 (1.00) | 36.00 | -1.00 (0.95) | 43.00 | -1.00 (0.93) | 36.00 | -0.86 (0.80) | 43.00 | n.a. (n.a.) | n.a. | n.a. (n.a.) | n.a. |
| Potthoff & Schienle, 2023 | Self-report | Subjective Sleep Quality | Change | -1.10 (0.64) | 36.00 | -1.09 (0.57) | 43.00 | -1.06 (0.63) | 36.00 | -1.00 (0.54) | 43.00 | n.a. (n.a.) | n.a. | n.a. (n.a.) | n.a. |
| Potthoff & Schienle, 2023 | Self-report | Use of sleep medication | Change | -0.05 (0.32) | 36.00 | -0.17 (0.62) | 43.00 | -0.56 (0.33) | 36.00 | -0.09 (0.43) | 43.00 | n.a. (n.a.) | n.a. | n.a. (n.a.) | n.a. |
| Rathschlag & Klatt, 2021a | Self-report | OLP+ well-being - ability to enjoy | Change | n.a. (n.a.) | n.a. | n.a. (n.a.) | n.a. | n.a. (n.a.) | n.a. | n.a. (n.a.) | n.a. | 0.13 (0.44) | 18.00 | 0.02 (0.37) | 16.00 |
| Rathschlag & Klatt, 2021a | Self-report | OLP+ well-being inner peace | Change | n.a. (n.a.) | n.a. | n.a. (n.a.) | n.a. | n.a. (n.a.) | n.a. | n.a. (n.a.) | n.a. | 0.47 (0.68) | 18.00 | 0.06 (0.44) | 16.00 |
| Rathschlag & Klatt, 2021a | Self-report | OLP+ well-being resilience | Change | n.a. (n.a.) | n.a. | n.a. (n.a.) | n.a. | n.a. (n.a.) | n.a. | n.a. (n.a.) | n.a. | 0.17 (0.56) | 18.00 | -0.09 (0.53) | 16.00 |
| Rathschlag & Klatt, 2021a | Self-report | OLP+ recovery and stress activation | Change | n.a. (n.a.) | n.a. | n.a. (n.a.) | n.a. | n.a. (n.a.) | n.a. | n.a. (n.a.) | n.a. | 0.31 (1.05) | 18.00 | 0.17 (1.60) | 16.00 |
| Rathschlag & Klatt, 2021a | Self-report | OLP+ recovery and stress emotional | Change | n.a. (n.a.) | n.a. | n.a. (n.a.) | n.a. | n.a. (n.a.) | n.a. | n.a. (n.a.) | n.a. | 0.17 (1.18) | 18.00 | 0.14 (0.73) | 16.00 |
| Rathschlag & Klatt, 2021a | Self-report | OLP+ recovery and stress mental | Change | n.a. (n.a.) | n.a. | n.a. (n.a.) | n.a. | n.a. (n.a.) | n.a. | n.a. (n.a.) | n.a. | 0.25 (0.92) | 18.00 | 0.14 (1.06) | 16.00 |
| Rathschlag & Klatt, 2021a | Self-report | OLP+ recovery and stress muscular | Change | n.a. (n.a.) | n.a. | n.a. (n.a.) | n.a. | n.a. (n.a.) | n.a. | n.a. (n.a.) | n.a. | -0.24 (1.62) | 18.00 | 0.11 (1.62) | 16.00 |
| Rathschlag & Klatt, 2021a | Self-report | OLP+ recovery and stress negative emotion | Change | n.a. (n.a.) | n.a. | n.a. (n.a.) | n.a. | n.a. (n.a.) | n.a. | n.a. (n.a.) | n.a. | 0.33 (1.27) | 18.00 | 0.06 (1.35) | 16.00 |
| Rathschlag & Klatt, 2021a | Self-report | OLP+ recovery and stress psysical | Change | n.a. (n.a.) | n.a. | n.a. (n.a.) | n.a. | n.a. (n.a.) | n.a. | n.a. (n.a.) | n.a. | 0.76 (1.19) | 18.00 | 0.16 (1.32) | 16.00 |
| Rathschlag & Klatt, 2021a | Self-report | OLP+ recovery and stress recovery | Change | n.a. (n.a.) | n.a. | n.a. (n.a.) | n.a. | n.a. (n.a.) | n.a. | n.a. (n.a.) | n.a. | 0.03 (1.29) | 18.00 | 0.05 (1.20) | 16.00 |
| Rathschlag & Klatt, 2021a | Self-report | OLP+ recovery and stress stress | Change | n.a. (n.a.) | n.a. | n.a. (n.a.) | n.a. | n.a. (n.a.) | n.a. | n.a. (n.a.) | n.a. | 0.18 (1.01) | 18.00 | -0.13 (1.66) | 16.00 |
| Rathschlag & Klatt, 2021a | Self-report | OLP+ well-being - vitality | Change | n.a. (n.a.) | n.a. | n.a. (n.a.) | n.a. | n.a. (n.a.) | n.a. | n.a. (n.a.) | n.a. | 0.31 (0.97) | 18.00 | -0.11 (0.58) | 16.00 |
| Rathschlag & Klatt, 2021b | Self-report | OLP+ well-being - ability to enjoy | Change | n.a. (n.a.) | n.a. | n.a. (n.a.) | n.a. | n.a. (n.a.) | n.a. | n.a. (n.a.) | n.a. | 0.24 (0.76) | 18.00 | 0.22 (0.64) | 19.00 |
| Rathschlag & Klatt, 2021b | Self-report | OLP+ well-being inner peace | Change | n.a. (n.a.) | n.a. | n.a. (n.a.) | n.a. | n.a. (n.a.) | n.a. | n.a. (n.a.) | n.a. | 0.43 (0.59) | 18.00 | 0.12 (0.45) | 19.00 |
| Rathschlag & Klatt, 2021b | Self-report | OLP+ well-being resilience | Change | n.a. (n.a.) | n.a. | n.a. (n.a.) | n.a. | n.a. (n.a.) | n.a. | n.a. (n.a.) | n.a. | 0.19 (0.79) | 18.00 | 0.11 (0.55) | 19.00 |
| Rathschlag & Klatt, 2021b | Self-report | OLP+ recovery and stress activation | Change | n.a. (n.a.) | n.a. | n.a. (n.a.) | n.a. | n.a. (n.a.) | n.a. | n.a. (n.a.) | n.a. | 0.33 (1.24) | 18.00 | -0.11 (0.94) | 19.00 |
| Rathschlag & Klatt, 2021b | Self-report | OLP+ recovery and stress emotional | Change | n.a. (n.a.) | n.a. | n.a. (n.a.) | n.a. | n.a. (n.a.) | n.a. | n.a. (n.a.) | n.a. | 0.36 (0.97) | 18.00 | -0.36 (0.57) | 19.00 |
| Rathschlag & Klatt, 2021b | Self-report | OLP+ recovery and stress mental | Change | n.a. (n.a.) | n.a. | n.a. (n.a.) | n.a. | n.a. (n.a.) | n.a. | n.a. (n.a.) | n.a. | 0.26 (1.06) | 18.00 | -0.04 (0.69) | 19.00 |
| Rathschlag & Klatt, 2021b | Self-report | OLP+ recovery and stress muscular | Change | n.a. (n.a.) | n.a. | n.a. (n.a.) | n.a. | n.a. (n.a.) | n.a. | n.a. (n.a.) | n.a. | -0.11 (2.14) | 18.00 | -0.42 (1.22) | 19.00 |
| Rathschlag & Klatt, 2021b | Self-report | OLP+ recovery and stress negative emotion | Change | n.a. (n.a.) | n.a. | n.a. (n.a.) | n.a. | n.a. (n.a.) | n.a. | n.a. (n.a.) | n.a. | 0.43 (1.21) | 18.00 | -0.51 (0.91) | 19.00 |
| Rathschlag & Klatt, 2021b | Self-report | OLP+ recovery and stress psysical | Change | n.a. (n.a.) | n.a. | n.a. (n.a.) | n.a. | n.a. (n.a.) | n.a. | n.a. (n.a.) | n.a. | 0.49 (1.04) | 18.00 | 0.16 (1.03) | 19.00 |
| Rathschlag & Klatt, 2021b | Self-report | OLP+ recovery and stress recovery | Change | n.a. (n.a.) | n.a. | n.a. (n.a.) | n.a. | n.a. (n.a.) | n.a. | n.a. (n.a.) | n.a. | 0.21 (0.68) | 18.00 | -0.11 (0.50) | 19.00 |
| Rathschlag & Klatt, 2021b | Self-report | OLP+ recovery and stress stress | Change | n.a. (n.a.) | n.a. | n.a. (n.a.) | n.a. | n.a. (n.a.) | n.a. | n.a. (n.a.) | n.a. | 0.35 (0.97) | 18.00 | 0.01 (1.25) | 19.00 |
| Rathschlag & Klatt, 2021b | Self-report | OLP+ well-being - vitality | Change | n.a. (n.a.) | n.a. | n.a. (n.a.) | n.a. | n.a. (n.a.) | n.a. | n.a. (n.a.) | n.a. | 0.54 (0.94) | 18.00 | 0.24 (0.59) | 19.00 |
| Rief & Glombiewski, 2012 | Objective | nasal passive+ active Open | Change | n.a. (n.a.) | n.a. | n.a. (n.a.) | n.a. | n.a. (n.a.) | n.a. | n.a. (n.a.) | n.a. | -0.82 (1.75) | 41.00 | -0.27 (1.48) | 20.00 |
| Schaefer et al., 2016 | Self-report | Allergic symptoms | Change | -3.55 (0.73) | 11.00 | -3.11 (0.66) | 14.00 | -2.67 (1.05) | 11.00 | -2.88 (0.77) | 14.00 | n.a. (n.a.) | n.a. | n.a. (n.a.) | n.a. |
| Schaefer et al., 2018 - | Self-report | Allergic symptoms OLP- vs CG (with&without) | Change | -3.22 (0.94) | 13.00 | -3.36 (1.03) | 20.00 | -2.79 (0.86) | 13.00 | -3.32 (1.02) | 20.00 | n.a. (n.a.) | n.a. | n.a. (n.a.) | n.a. |
| Schaefer et al., 2018 + | Self-report | Allergic symptoms OLP+ vs CG (with&without) | Change | -3.31 (0.70) | 13.00 | -3.36 (1.03) | 20.00 | -2.53 (0.63) | 13.00 | -3.32 (1.02) | 20.00 | n.a. (n.a.) | n.a. | n.a. (n.a.) | n.a. |
| Schaefer et al., 2019 | Self-report | Change in self-management-abilities | Change | n.a. (n.a.) | n.a. | n.a. (n.a.) | n.a. | n.a. (n.a.) | n.a. | n.a. (n.a.) | n.a. | 15.77 (31.71) | 31.00 | 2.48 (15.46) | 27.00 |
| Schaefer et al., 2019 | Self-report | Change in test anxiety | Change | n.a. (n.a.) | n.a. | n.a. (n.a.) | n.a. | n.a. (n.a.) | n.a. | n.a. (n.a.) | n.a. | 4.39 (9.35) | 31.00 | 0.07 (6.00) | 27.00 |
| Schaefer et al., 2021 | Self-report | PANAS (mood and subjective stress) | Change | n.a. (n.a.) | n.a. | n.a. (n.a.) | n.a. | n.a. (n.a.) | n.a. | n.a. (n.a.) | n.a. | -0.05 (0.45) | 24.00 | -0.12 (0.46) | 29.00 |
| Schaefer et al., 2021 | Self-report | STAI-S (state anxiety) | Change | n.a. (n.a.) | n.a. | n.a. (n.a.) | n.a. | n.a. (n.a.) | n.a. | n.a. (n.a.) | n.a. | -1.17 (5.21) | 24.00 | -1.90 (7.34) | 29.00 |
| Schaefer et al., 2021 | Self-report | VAS (subjectively perceived stressfulness; current stress) | Change | n.a. (n.a.) | n.a. | n.a. (n.a.) | n.a. | n.a. (n.a.) | n.a. | n.a. (n.a.) | n.a. | -31.29 (35.07) | 24.00 | -30.76 (34.60) | 29.00 |
| Schaefer et al., 2023a | Self-report | Allergic symptoms: severity | Change | n.a. (n.a.) | n.a. | n.a. (n.a.) | n.a. | n.a. (n.a.) | n.a. | n.a. (n.a.) | n.a. | 1.20 (2.41) | 22.00 | 0.42 (2.39) | 26.00 |
| Schaefer et al., 2023a | Self-report | Allergic symptoms: severity | Change | n.a. (n.a.) | n.a. | n.a. (n.a.) | n.a. | n.a. (n.a.) | n.a. | n.a. (n.a.) | n.a. | 1.36 (2.03) | 22.00 | 0.15 (2.32) | 26.00 |
| Schaefer et al., 2023b | Self-report | Emotional distress | Post-only | n.a. (n.a.) | n.a. | n.a. (n.a.) | n.a. | n.a. (n.a.) | n.a. | n.a. (n.a.) | n.a. | -4.85 (1.49) | 58.00 | -5.51 (1.56) | 54.00 |
| Schaefer et al., 2023c | Self-report | Emotional distress | Post-only | n.a. (n.a.) | n.a. | n.a. (n.a.) | n.a. | n.a. (n.a.) | n.a. | n.a. (n.a.) | n.a. | -1.60 (0.76) | 23.00 | -2.04 (0.62) | 21.00 |
| Schienle & Jurinec, 2022 | Self-report | Depressive symptoms | Change | n.a. (n.a.) | n.a. | n.a. (n.a.) | n.a. | n.a. (n.a.) | n.a. | n.a. (n.a.) | n.a. | 7.93 (2.26) | 30.00 | 5.47 (3.19) | 30.00 |
| Schienle & Unger, 2021 | Self-report | Exercises quantity:PMR | Post-only | n.a. (n.a.) | n.a. | n.a. (n.a.) | n.a. | n.a. (n.a.) | n.a. | n.a. (n.a.) | n.a. | 9.75 (4.10) | 68.00 | 8.15 (4.40) | 80.00 |
| Schienle & Unger, 2023 | Self-report | Acts of kindness | Post-only | n.a. (n.a.) | n.a. | n.a. (n.a.) | n.a. | n.a. (n.a.) | n.a. | n.a. (n.a.) | n.a. | 5.83 (1.55) | 71.00 | 5.19 (1.82) | 89.00 |
| Schienle et al., 2022 | Objective | Early LPP frontal angry | Post-only | n.a. (n.a.) | n.a. | n.a. (n.a.) | n.a. | n.a. (n.a.) | n.a. | n.a. (n.a.) | n.a. | -0.27 (3.46) | 52.00 | -1.65 (2.79) | 51.00 |
| Schienle et al., 2022 | Objective | Late LPP frontal angry | Post-only | n.a. (n.a.) | n.a. | n.a. (n.a.) | n.a. | n.a. (n.a.) | n.a. | n.a. (n.a.) | n.a. | -0.76 (2.38) | 52.00 | -1.95 (1.86) | 51.00 |
| Schienle et al., 2022 | Objective | Early LPP centroparietal angry | Post-only | n.a. (n.a.) | n.a. | n.a. (n.a.) | n.a. | n.a. (n.a.) | n.a. | n.a. (n.a.) | n.a. | -2.74 (3.03) | 52.00 | -4.49 (3.21) | 51.00 |
| Schienle et al., 2022 | Objective | Late LPP centroparietal angry | Post-only | n.a. (n.a.) | n.a. | n.a. (n.a.) | n.a. | n.a. (n.a.) | n.a. | n.a. (n.a.) | n.a. | -1.60 (2.38) | 52.00 | -2.62 (1.86) | 51.00 |
| Schienle et al., 2022 | Self-report | Anger | Post-only | n.a. (n.a.) | n.a. | n.a. (n.a.) | n.a. | n.a. (n.a.) | n.a. | n.a. (n.a.) | n.a. | -70.07 (15.41) | 52.00 | -77.41 (13.98) | 51.00 |
| Schienle et al., 2022 | Self-report | Arousal | Post-only | n.a. (n.a.) | n.a. | n.a. (n.a.) | n.a. | n.a. (n.a.) | n.a. | n.a. (n.a.) | n.a. | -16.46 (17.47) | 52.00 | -25.11 (18.89) | 51.00 |
| Schienle et al., 2023 | Self-report | Disgust | Post-only | n.a. (n.a.) | n.a. | n.a. (n.a.) | n.a. | n.a. (n.a.) | n.a. | n.a. (n.a.) | n.a. | -5.34 (1.45) | 48.00 | -5.88 (1.34) | 49.00 |
| Schneider et al., 2006 | Objective | Diastolic blood pressure (mmHg) | Change | 70.30 (9.30) | 15.00 | 65.10 (9.35) | 15.00 | 69.30 (9.30) | 15.00 | 64.30 (10.20) | 15.00 | n.a. (n.a.) | n.a. | n.a. (n.a.) | n.a. |
| Schneider et al., 2006 | Objective | Heart rate (Beats per Minute) | Change | 78.40 (7.10) | 15.00 | 64.30 (10.20) | 15.00 | 73.60 (8.20) | 15.00 | 70.20 (4.80) | 15.00 | n.a. (n.a.) | n.a. | n.a. (n.a.) | n.a. |
| Schneider et al., 2006 | Objective | Reaction time (ms) | Change | -245.00 (57.00) | 15.00 | -222.00 (40.00) | 15.00 | -240.00 (54.00) | 15.00 | -223.00 (27.00) | 15.00 | n.a. (n.a.) | n.a. | n.a. (n.a.) | n.a. |
| Schneider et al., 2006 | Objective | Systolic blood pressure (mmHg) | Change | 113.20 (12.50) | 15.00 | 110.60 (8.70) | 15.00 | 111.70 (12.30) | 15.00 | 106.20 (11.30) | 15.00 | n.a. (n.a.) | n.a. | n.a. (n.a.) | n.a. |
| Schneider et al., 2006 | Self-report | Alertness | Change | 30.30 (7.40) | 15.00 | 25.90 (7.40) | 15.00 | 27.50 (7.40) | 15.00 | 25.10 (7.40) | 15.00 | n.a. (n.a.) | n.a. | n.a. (n.a.) | n.a. |
| Schneider et al., 2006 | Self-report | Mood | Change | 31.10 (7.40) | 15.00 | 31.30 (6.00) | 15.00 | 31.80 (5.40) | 15.00 | 32.80 (5.10) | 15.00 | n.a. (n.a.) | n.a. | n.a. (n.a.) | n.a. |
| Schneider et al., 2006 | Self-report | Calmness | Post-only | -31.40 (4.60) | 15.00 | -31.80 (5.20) | 15.00 | -31.60 (4.00) | 15.00 | -33.50 (4.90) | 15.00 | n.a. (n.a.) | n.a. | n.a. (n.a.) | n.a. |
| Sezer et al., 2022 | Self-report | Guilt | Change | -2.06 (1.00) | 35.00 | -1.92 (0.70) | 39.00 | -2.18 (0.93) | 35.00 | -2.36 (0.94) | 39.00 | n.a. (n.a.) | n.a. | n.a. (n.a.) | n.a. |
| Stumpp et al., 2023 | Self-report | Hurt feelings | Change | n.a. (n.a.) | n.a. | n.a. (n.a.) | n.a. | n.a. (n.a.) | n.a. | n.a. (n.a.) | n.a. | -4.57 (1.60) | 21.00 | -6.13 (2.39) | 15.00 |
| Stumpp et al., 2023 | Self-report | Need threat | Post-only | n.a. (n.a.) | n.a. | n.a. (n.a.) | n.a. | n.a. (n.a.) | n.a. | n.a. (n.a.) | n.a. | -6.14 (0.80) | 21.00 | -6.52 (1.58) | 15.00 |
| Urroz et al., 2016 | Objective | Blood lactate (15 min) | Change | -5.06 (3.30) | 12.00 | -4.51 (3.33) | 12.00 | -4.62 (2.76) | 12.00 | -3.92 (2.78) | 12.00 | n.a. (n.a.) | n.a. | n.a. (n.a.) | n.a. |
| Urroz et al., 2016 | Objective | Blood lactate (30 min) | Change | -5.06 (3.30) | 12.00 | -4.51 (3.33) | 12.00 | -3.21 (2.34) | 12.00 | -2.33 (2.36) | 12.00 | n.a. (n.a.) | n.a. | n.a. (n.a.) | n.a. |
| Urroz et al., 2016 | Objective | Blood lactate (45 min) | Change | -5.06 (3.30) | 12.00 | -4.51 (3.33) | 12.00 | -1.82 (1.97) | 12.00 | -1.20 (1.99) | 12.00 | n.a. (n.a.) | n.a. | n.a. (n.a.) | n.a. |
| Urroz et al., 2016 | Objective | Blood lactate (60 min) | Change | -5.06 (3.30) | 12.00 | -4.51 (3.33) | 12.00 | -1.45 (1.45) | 12.00 | -0.91 (1.46) | 12.00 | n.a. (n.a.) | n.a. | n.a. (n.a.) | n.a. |
| Urroz et al., 2016 | Objective | Diastolic blood pressure (10 min) | Change | -78.00 (6.00) | 12.00 | -76.00 (6.00) | 12.00 | -73.00 (7.00) | 12.00 | -72.00 (7.00) | 12.00 | n.a. (n.a.) | n.a. | n.a. (n.a.) | n.a. |
| Urroz et al., 2016 | Objective | Diastolic blood pressure (20 min) | Change | -78.00 (6.00) | 12.00 | -76.00 (6.00) | 12.00 | -73.00 (7.00) | 12.00 | -72.00 (7.00) | 12.00 | n.a. (n.a.) | n.a. | n.a. (n.a.) | n.a. |
| Urroz et al., 2016 | Objective | Diastolic blood pressure (30 min) | Change | -78.00 (6.00) | 12.00 | -76.00 (6.00) | 12.00 | -75.00 (7.00) | 12.00 | -73.00 (7.00) | 12.00 | n.a. (n.a.) | n.a. | n.a. (n.a.) | n.a. |
| Urroz et al., 2016 | Objective | Diastolic blood pressure (40 min) | Change | -78.00 (6.00) | 12.00 | -76.00 (6.00) | 12.00 | -73.00 (7.00) | 12.00 | -73.00 (7.00) | 12.00 | n.a. (n.a.) | n.a. | n.a. (n.a.) | n.a. |
| Urroz et al., 2016 | Objective | Diastolic blood pressure (50 min) | Change | -78.00 (6.00) | 12.00 | -76.00 (6.00) | 12.00 | -73.00 (7.00) | 12.00 | -74.00 (7.00) | 12.00 | n.a. (n.a.) | n.a. | n.a. (n.a.) | n.a. |
| Urroz et al., 2016 | Objective | Diastolic blood pressure (60 min) | Change | -78.00 (6.00) | 12.00 | -76.00 (6.00) | 12.00 | -74.00 (7.00) | 12.00 | -73.00 (7.00) | 12.00 | n.a. (n.a.) | n.a. | n.a. (n.a.) | n.a. |
| Urroz et al., 2016 | Objective | Heart rate (10 min) | Change | -176.00 (12.00) | 12.00 | -184.00 (12.00) | 12.00 | -89.00 (12.00) | 12.00 | -97.00 (12.00) | 12.00 | n.a. (n.a.) | n.a. | n.a. (n.a.) | n.a. |
| Urroz et al., 2016 | Objective | Heart rate (20 min) | Change | -176.00 (12.00) | 12.00 | -184.00 (12.00) | 12.00 | -81.00 (10.00) | 12.00 | -94.00 (10.00) | 12.00 | n.a. (n.a.) | n.a. | n.a. (n.a.) | n.a. |
| Urroz et al., 2016 | Objective | Heart rate (30 min) | Change | -176.00 (12.00) | 12.00 | -184.00 (12.00) | 12.00 | -78.00 (10.00) | 12.00 | -86.00 (10.00) | 12.00 | n.a. (n.a.) | n.a. | n.a. (n.a.) | n.a. |
| Urroz et al., 2016 | Objective | Heart rate (40 min) | Change | -176.00 (12.00) | 12.00 | -184.00 (12.00) | 12.00 | -75.00 (12.00) | 12.00 | -84.00 (12.00) | 12.00 | n.a. (n.a.) | n.a. | n.a. (n.a.) | n.a. |
| Urroz et al., 2016 | Objective | Heart rate (50 min) | Change | -176.00 (12.00) | 12.00 | -184.00 (12.00) | 12.00 | -71.00 (9.00) | 12.00 | -80.00 (9.00) | 12.00 | n.a. (n.a.) | n.a. | n.a. (n.a.) | n.a. |
| Urroz et al., 2016 | Objective | Heart rate (60 min) | Change | -176.00 (12.00) | 12.00 | -184.00 (12.00) | 12.00 | -69.00 (9.00) | 12.00 | -79.00 (9.00) | 12.00 | n.a. (n.a.) | n.a. | n.a. (n.a.) | n.a. |
| Urroz et al., 2016 | Objective | Respiratory rate (10 min) | Change | -39.00 (9.00) | 12.00 | -37.00 (9.00) | 12.00 | -23.00 (27.00) | 12.00 | -22.00 (27.00) | 12.00 | n.a. (n.a.) | n.a. | n.a. (n.a.) | n.a. |
| Urroz et al., 2016 | Objective | Respiratory rate (20 min) | Change | -39.00 (9.00) | 12.00 | -37.00 (9.00) | 12.00 | -20.00 (4.00) | 12.00 | -18.00 (4.00) | 12.00 | n.a. (n.a.) | n.a. | n.a. (n.a.) | n.a. |
| Urroz et al., 2016 | Objective | Respiratory rate (30 min) | Change | -39.00 (9.00) | 12.00 | -37.00 (9.00) | 12.00 | -19.00 (4.00) | 12.00 | -17.00 (4.00) | 12.00 | n.a. (n.a.) | n.a. | n.a. (n.a.) | n.a. |
| Urroz et al., 2016 | Objective | Respiratory rate (40 min) | Change | -39.00 (9.00) | 12.00 | -37.00 (9.00) | 12.00 | -18.00 (4.00) | 12.00 | -16.00 (4.00) | 12.00 | n.a. (n.a.) | n.a. | n.a. (n.a.) | n.a. |
| Urroz et al., 2016 | Objective | Respiratory rate (50 min) | Change | -39.00 (9.00) | 12.00 | -37.00 (9.00) | 12.00 | -18.00 (4.00) | 12.00 | -15.00 (4.00) | 12.00 | n.a. (n.a.) | n.a. | n.a. (n.a.) | n.a. |
| Urroz et al., 2016 | Objective | Respiratory rate (60 min) | Change | -39.00 (9.00) | 12.00 | -37.00 (9.00) | 12.00 | -17.00 (4.00) | 12.00 | -16.00 (4.00) | 12.00 | n.a. (n.a.) | n.a. | n.a. (n.a.) | n.a. |
| Urroz et al., 2016 | Objective | Systolic blood pressure (10 min) | Change | -144.00 (13.00) | 12.00 | -147.00 (14.00) | 12.00 | -117.00 (9.00) | 12.00 | -120.00 (9.00) | 12.00 | n.a. (n.a.) | n.a. | n.a. (n.a.) | n.a. |
| Urroz et al., 2016 | Objective | Systolic blood pressure (20 min) | Change | -144.00 (13.00) | 12.00 | -147.00 (14.00) | 12.00 | -113.00 (8.00) | 12.00 | -116.00 (8.00) | 12.00 | n.a. (n.a.) | n.a. | n.a. (n.a.) | n.a. |
| Urroz et al., 2016 | Objective | Systolic blood pressure (30 min) | Change | -144.00 (13.00) | 12.00 | -147.00 (14.00) | 12.00 | -112.00 (9.00) | 12.00 | -114.00 (9.00) | 12.00 | n.a. (n.a.) | n.a. | n.a. (n.a.) | n.a. |
| Urroz et al., 2016 | Objective | Systolic blood pressure (40 min) | Change | -144.00 (13.00) | 12.00 | -147.00 (14.00) | 12.00 | -110.00 (9.00) | 12.00 | -112.00 (9.00) | 12.00 | n.a. (n.a.) | n.a. | n.a. (n.a.) | n.a. |
| Urroz et al., 2016 | Objective | Systolic blood pressure (50 min) | Change | -144.00 (13.00) | 12.00 | -147.00 (14.00) | 12.00 | -109.00 (9.00) | 12.00 | -111.00 (9.00) | 12.00 | n.a. (n.a.) | n.a. | n.a. (n.a.) | n.a. |
| Urroz et al., 2016 | Objective | Systolic blood pressure (60 min) | Change | -144.00 (13.00) | 12.00 | -147.00 (14.00) | 12.00 | -109.00 (9.00) | 12.00 | -110.00 (9.00) | 12.00 | n.a. (n.a.) | n.a. | n.a. (n.a.) | n.a. |
| Urroz et al., 2016 | Objective | Volume of oxygen consumption (10 min) | Change | -2558.00 (673.00) | 12.00 | -2356.00 (679.00) | 12.00 | -402.00 (111.00) | 12.00 | -399.00 (112.00) | 12.00 | n.a. (n.a.) | n.a. | n.a. (n.a.) | n.a. |
| Urroz et al., 2016 | Objective | Volume of oxygen consumption (20 min) | Change | -2558.00 (673.00) | 12.00 | -2356.00 (679.00) | 12.00 | -343.00 (101.00) | 12.00 | -343.00 (102.00) | 12.00 | n.a. (n.a.) | n.a. | n.a. (n.a.) | n.a. |
| Urroz et al., 2016 | Objective | Volume of oxygen consumption (30 min) | Change | -2558.00 (673.00) | 12.00 | -2356.00 (679.00) | 12.00 | -342.00 (104.00) | 12.00 | -325.00 (105.00) | 12.00 | n.a. (n.a.) | n.a. | n.a. (n.a.) | n.a. |
| Urroz et al., 2016 | Objective | Volume of oxygen consumption (40 min) | Change | -2558.00 (673.00) | 12.00 | -2356.00 (679.00) | 12.00 | -329.00 (101.00) | 12.00 | -330.00 (102.00) | 12.00 | n.a. (n.a.) | n.a. | n.a. (n.a.) | n.a. |
| Urroz et al., 2016 | Objective | Volume of oxygen consumption (50 min) | Change | -2558.00 (673.00) | 12.00 | -2356.00 (679.00) | 12.00 | -305.00 (104.00) | 12.00 | -274.00 (105.00) | 12.00 | n.a. (n.a.) | n.a. | n.a. (n.a.) | n.a. |
| Urroz et al., 2016 | Objective | Volume of oxygen consumption (60 min) | Change | -2558.00 (673.00) | 12.00 | -2356.00 (679.00) | 12.00 | -296.00 (104.00) | 12.00 | -280.00 (105.00) | 12.00 | n.a. (n.a.) | n.a. | n.a. (n.a.) | n.a. |
| Walach et al., 2001 | Objective | Diastolic blood pressure (mmHg) | Change | n.a. (n.a.) | n.a. | n.a. (n.a.) | n.a. | n.a. (n.a.) | n.a. | n.a. (n.a.) | n.a. | -7.90 (7.70) | 41.00 | -3.50 (2.70) | 37.00 |
| Walach et al., 2001 | Objective | Heart rate (bpm) | Change | n.a. (n.a.) | n.a. | n.a. (n.a.) | n.a. | n.a. (n.a.) | n.a. | n.a. (n.a.) | n.a. | -6.20 (8.30) | 41.00 | -4.10 (5.20) | 37.00 |
| Walach et al., 2001 | Objective | Systolic blood pressure (mmHg) | Change | n.a. (n.a.) | n.a. | n.a. (n.a.) | n.a. | n.a. (n.a.) | n.a. | n.a. (n.a.) | n.a. | -2.30 (5.60) | 41.00 | -2.70 (4.90) | 37.00 |
| Walach et al., 2001 | Self-report | Basle Well Being Scale | Change | n.a. (n.a.) | n.a. | n.a. (n.a.) | n.a. | n.a. (n.a.) | n.a. | n.a. (n.a.) | n.a. | 3.60 (8.10) | 41.00 | -0.80 (9.30) | 37.00 |
| Winkler et al., 2023 | Self-report | Depression nasal active+ nasal passive+ pills | Change | -7.45 (5.26) | 132.00 | -6.73 (4.30) | 45.00 | -5.10 (4.79) | 132.00 | -5.91 (4.89) | 45.00 | n.a. (n.a.) | n.a. | n.a. (n.a.) | n.a. |
| Winkler et al., 2023 | Self-report | Stress nasal active+ nasal passive+ pills | Change | -23.99 (5.53) | 132.00 | -24.56 (4.44) | 45.00 | -19.02 (6.61) | 132.00 | -20.98 (5.59) | 45.00 | n.a. (n.a.) | n.a. | n.a. (n.a.) | n.a. |
| Yennurajalingam et al., 2022 | Self-report | Cancer related fatigue | Change | n.a. (n.a.) | n.a. | n.a. (n.a.) | n.a. | n.a. (n.a.) | n.a. | n.a. (n.a.) | n.a. | 6.60 (7.60) | 42.00 | 2.10 (9.40) | 42.00 |
| Zhou et al., 2019 | Self-report | Cancer related fatigue | Change | 28.40 (9.59) | 20.00 | 25.80 (9.33) | 20.00 | 32.70 (11.10) | 20.00 | 27.00 (10.81) | 20.00 | n.a. (n.a.) | n.a. | n.a. (n.a.) | n.a. |

*Note*. ^a^ OLP = open-label placebo. ^b^ SD = standard deviation. ^c^ N = number. ^d^CG = control group. ^e^ n.a. = not available. ^f ‘^–‘ = without suggestive treatment rationale. ^g^ ‘+’ = with suggestive treatment rationale.

## References

1. Ashar, Y., Perlis, R., Liston, C., Gunning, F. & Wager, T. Effects of pain reprocessing therapy on attributed causes of chronic back pain. *The Journal of Pain* **23**, 27–28 (2022).

2. Bandak, E. *et al.* Exercise and education versus saline injections for knee osteoarthritis: a randomised controlled equivalence trial. *Annals of the Rheumatic Diseases* **81**, 537–543 (2022).

3. Braescher, A.-K., Ferti, I.-E. & Witthöft, M. Open-label placebo effects on psychological and physical well-being: a conceptual replication study. (2022) doi:10.32872/cpe.7679.

4. Bush, N. J., Boissoneault, J., Letzen, J., Staud, R. & Robinson, M. E. Task-dependent functional connectivity of pain is associated with the magnitude of placebo analgesia in pain-free individuals. *Eur J Pain* **27**, 1023–1035 (2023).

5. Bush, N., Robinson, M., Bryan, M., Staud, R. & Boissoneault, J. Task-dependent functional connectivity of pain-related brain regions is related to magnitude of placebo analgesia. *The Journal of Pain* **22**, 603 (2021).

6. Carvalho, C. *et al.* Open-label placebo for chronic low back pain: a 5-year follow-up. *Pain* **162**, 1521–1527 (2021).

7. Choi, D.-H., Lee, I.-S. & Chae, Y. Open label placebo: pill and needle. *Integr. Med. Res.* **9**, (2020).

8. Davies, J. N., Sharpe, L., Day, M. A. & Colagiuri, B. How do placebo effects contribute to mindfulness-based analgesia? Probing acute pain effects and interactions using a randomized balanced placebo design. *Pain* **163**, 1967–1977 (2022).

9. De Vita, M. J. *et al.* The effects of cannabidiol and analgesic expectancies on experimental pain reactivity in healthy adults: a balanced placebo design trial. *Exp. Clin. Psychopharmacol.* **30**, 536–546 (2022).

10. Disley, N., Kola-Palmer, S. & Retzler, C. A comparison of open-label and deceptive placebo analgesia in a healthy sample. *Journal of Psychosomatic Research* **140**, (2021).

11. Frommelt, T., Traykova, M., Platt, B. & Wittekind, C. E. The influence of outcome expectancy on interpretation bias training in social anxiety: an experimental pilot study. *Pilot Feasibility Stud* **9**, 144 (2023).

12. Guevarra, D., Kross, E. & Moser, J. S. Outsourcing affect regulation to non-deceptive placebos. *Int. J. Psychophysiol.* **188**, 36 (2023).

13. Haas, J. W., Winkler, A., Rheker, J., Doering, B. K. & Rief, W. No open-label placebo effect in insomnia? Lessons learned from an experimental trial. *J. Psychosom. Res.* **158**, (2022).

14. Hamberger, J. *et al.* Health economic evaluation of an open-label placebo intervention in patients with functional post-covid syndrome. *Zeitschrift Fur Psychosomatische Medizin Und Psychotherapie* **69**, 133–134 (2023).

15. Henriksen, M. *et al.* Exercise and education vs intra-articular saline for knee osteoarthritis: a 1-year follow-up of a randomized trial. *Osteoarthritis Cartilage* **31**, 627–635 (2023).

16. Hoenemeyer, T. W. *et al.* An exploratory analysis of the association between catechol-o-methyltransferase and response to a randomized open-label placebo treatment for cancer-related fatigue. *Front. Psychiatry* **12**, (2021).

17. Kleine-Borgmann, J., Dietz, T.-N., Schmidt, K. & Bingel, U. No long-term effects after a 3-week open-label placebo treatment for chronic low back pain: a 3-year follow-up of a randomized controlled trial. *Pain* **164**, 645–652 (2023).

18. Lee, S., Choi, D.-H., Hong, M., Lee, I.-S. & Chae, Y. Open-label placebo treatment for experimental pain: a randomized-controlled trial with placebo acupuncture and placebo pills. *J. Integr. Complement. Med.* **28**, 136–145 (2022).

19. Meeuwis, S. H., Van Middendorp, H., Lavrijsen, A. P. M., Veldhuijzen, D. S. & Evers, A. W. M. Open- And closed-label placebo and nocebo suggestions about a sham transdermal patch. *Psychosom. Med.* **83**, 33–42 (2021).

20. Meijer, S. *et al.* Efficacy of open-label counterconditioning for reducing nocebo effects on pressure pain. *European Journal of Pain* **27**, 831–847 (2023).

21. Nurko, S. *et al.* 814 placebo without deception is effective in the treatment of children with functional gastrointestinal disorders (fgids). **158**, S‐161 (2020).

22. Pan, Y. *et al.* Open-label placebos for menopausal hot flushes: A randomized controlled trial. *Sci Rep* **10**, 20090 (2020).

23. Ort, S. *et al.* The effects of pilates and open-label placebo on severe primary dysmenorrhea-a randomized controlled pilot study. *Psychosom. Med.* **85**, A28 (2023).

24. van Lennep, J. (Hans) P. A. *et al.* The optimal learning cocktail for placebo analgesia: a randomized controlled trial comparing individual and combined techniques. *The Journal of Pain* (2023) doi:10.1016/j.jpain.2023.07.009.

25. Zaworski, K., Kadłubowska, M. & Baj-Korpak, J. Impact of verbal suggestions on finger flexor activation and strength in healthy individuals. *Med Sci Monit* **29**, e941548-1-e941548-10 (2023).

26. Barnes, K., Yu, A., Josupeit, J. & Colagiuri, B. Deceptive but not open label placebos attenuate motion-induced nausea. *Journal of Psychosomatic Research* **125**, 109808 (2019).

27. Spille, L., Fendel, J. C., Seuling, P. D., Göritz, A. S. & Schmidt, S. Open-label placebos—a systematic review and meta-analysis of experimental studies with non-clinical samples. *Sci Rep* **13**, 3640 (2023).

28. Buergler, S., Sezer, D., Gaab, J. & Locher, C. The roles of expectation, comparator, administration route, and population in open-label placebo effects: A network meta-analysis. *Sci Rep* **13**, 11827 (2023).

29. Haas, J. W., Rief, W., Glombiewski, J. A., Winkler, A. & Doering, B. K. Expectation-induced placebo effect on acute sadness in women with major depression: An experimental investigation. *Journal of Affective Disorders* **274**, 920–928 (2020).

30. Guevarra, D. A., Moser, J. S., Wager, T. D. & Kross, E. Placebos without deception reduce self-report and neural measures of emotional distress. *Nat Commun* **11**, 3785 (2020).

31. Rathschlag, M. & Klatt, S. Open-label placebo interventions with drinking water and their influence on perceived physical and mental well-being. *Front Psychol* **12**, 658275 (2021).

32. Schaefer, M., Kühnel, A., Schweitzer, F., Enge, S. & Gärtner, M. Neural underpinnings of open-label placebo effects in emotional distress. *Neuropsychopharmacology* **48**, 560–566 (2023).

33. von Wernsdorff, M., Loef, M., Tuschen-Caffier, B. & Schmidt, S. Effects of open-label placebos in clinical trials: A systematic review and meta-analysis. *Sci Rep* **11**, 3855 (2021).

34. Disley, N., Kola-Palmer, S. & Retzler, C. A comparison of open-label and deceptive placebo analgesia in a healthy sample. *JOURNAL OF PSYCHOSOMATIC RESEARCH* **140**, (2021).

35. De Vita, M. J. *et al.* The effects of cannabidiol and analgesic expectancies on experimental pain reactivity in healthy adults: A balanced placebo design trial. *Exp Clin Psychopharmacol* **30**, 536–546 (2022).

36. Sandler, A. D., Glesne, C. E. & Bodfish, J. W. Conditioned placebo dose reduction: A new treatment in Attention-Deficit Hyperactivity Disorder? *Journal of Developmental & Behavioral Pediatrics* **31**, 369 (2010).
